# Supplementary material for: Self-Healing Iron Oxide Polyelectrolyte Nanocomposites: Influence of Particle Agglomeration and Water on Mechanical Properties
Source: Nanomaterials (Basel). 2023 Nov 21;13(23):2983. doi: 10.3390/nano13232983 (PMC10708423; doi:10.3390/nano13232983)
Supplement: Supplementary file 1 [file nanomaterials-13-02983-s001.zip › nanomaterials-2721002-supplementary.pdf]

Supporting Information

Self-Healing Iron Oxide Polyelectrolyte Nanocomposites: Influence of Particle Agglomeration and Water on Mechanical Properties

Bastian Oberhausen [1], Ajda Plohl [1], Bart-Jan Niebuur [2], Stefan Diebels [3], Anne Jung [4], Tobias Kraus [2,5] and Guido Kickelbick [1,*]

[1] Inorganic Solid-State Chemistry, Saarland University, Campus, Building C4 1, 66123 Saarbrücken, Germany; bastian.oberhausen@uni-saarland.de (B.O.); ajda.plohl@uni-saarland.de (A.P.)
[2] INM—Leibniz-Institute for New Materials, Campus, D2 2, 66123 Saarbrücken, Germany; tobias.kraus@leibniz-inm.de (T.K.)
[3] Applied Mechanics, Saarland University, Campus, Building A4 2, 66123 Saarbrücken, Germany; s.diebels@mx.uni-saarland.de
[4] Protective Systems, Helmut-Schmidt-University/University of the Federal Armed Forces Hamburg, Holstenhofweg 85, 22043 Hamburg, Germany; anne.jung@hsu-hh.de
[5] Colloid and Interface Chemistry, Saarland University, Campus, Building D2 2, 66123 Saarbrücken, Germany
* Correspondence: guido.kickelbick@uni-saarland.de; Tel.: +43-681-302-70651

Content

1. Synthesis and characterization of the cationic organophosphorus coupling molecule and its precursors....... 2
2. Synthesis and characterization of OA@Fe_XO_Y and $^{0.200}$P@Fe_XO_Y....... 8
3. Synthesis and characterization of SMBS....... 11
4. Optimization of the polymerization procedure....... 13
5. Water adsorption studies of the synthesized polymers and composites....... 19
6. Rheology measurements of the DOW Corning OE6630 reference samples....... 21
7. Rheology measurements of the polymer samples....... 22
8. TGA measurements of at different drying times at 80 °C....... 26
9. Tensile testing of the Polymer and composite samples....... 27
10. Temperature Stability of the Polymer Matrix....... 29
11. Results of the SAXS Measurements....... 30
12. Microscopy images of the-self healing tests....... 32

1. Synthesis and characterization of the cationic organophosphorus coupling molecule and its precursors.

An overview of the synthesis of *N*,*N*,*N*-trimethyl-6-phosphonohexan-1-ammonium bromide is shown in Scheme S1. The synthesis was carried out according to a previously described procedure.[1]

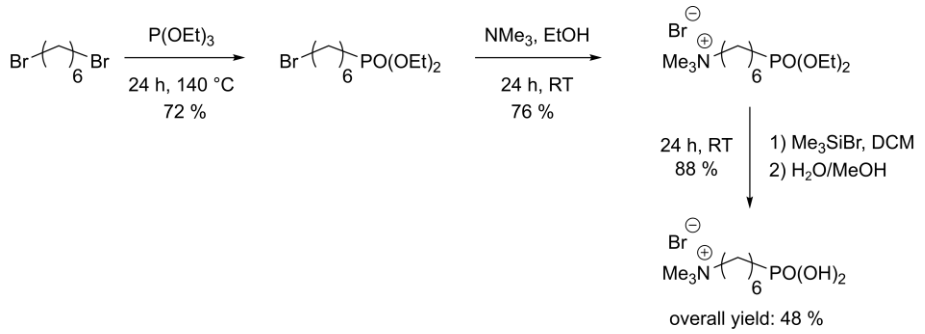

Scheme S1: Synthesis route for *N*,*N*,*N*-trimethyl-6-phosphonohexan-1-ammonium bromide.

Diethyl(6-bromohexyl) phosphonate: 24.40 g (100 mmol) 1,6-dibromohexane was heated to 150 °C. Within one hour, 16.60 g (100 mmol) triethyl phosphite was added. The reaction mixture was kept at 150 °C for a further 5 hours and then cooled to room temperature. The crude product was fractionally distilled at reduced pressure (Bpt 0.08 mbar: 123 °C).

Yield: 19.97 g, 66 mmol, 66%, colorless oil.

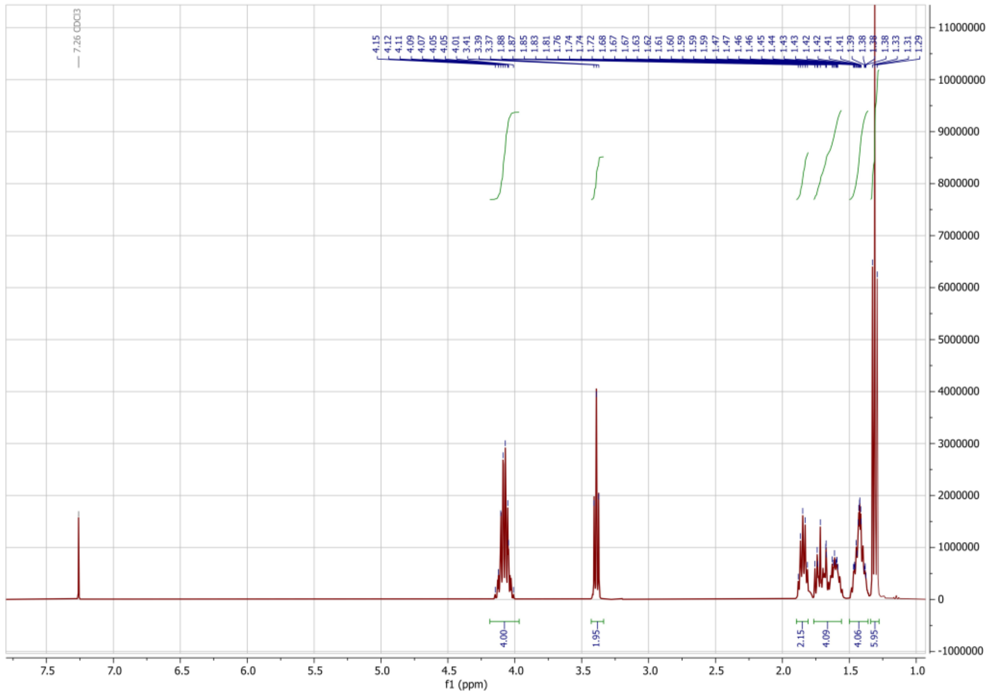

Figure S1: ^{1}H NMR spectrum of diethyl(6-bromohexyl) phosphonate.

^{1}H NMR δ ($CDCl_3$): 4.01-4.15 (4H, O-CH_2-CH_3), 3.37-3.41 (2H, CH_2-Br), 1.38-1.88 (10H, CH_2), 1.29-1.33 (6H, O-CH_2-CH_3) ppm.

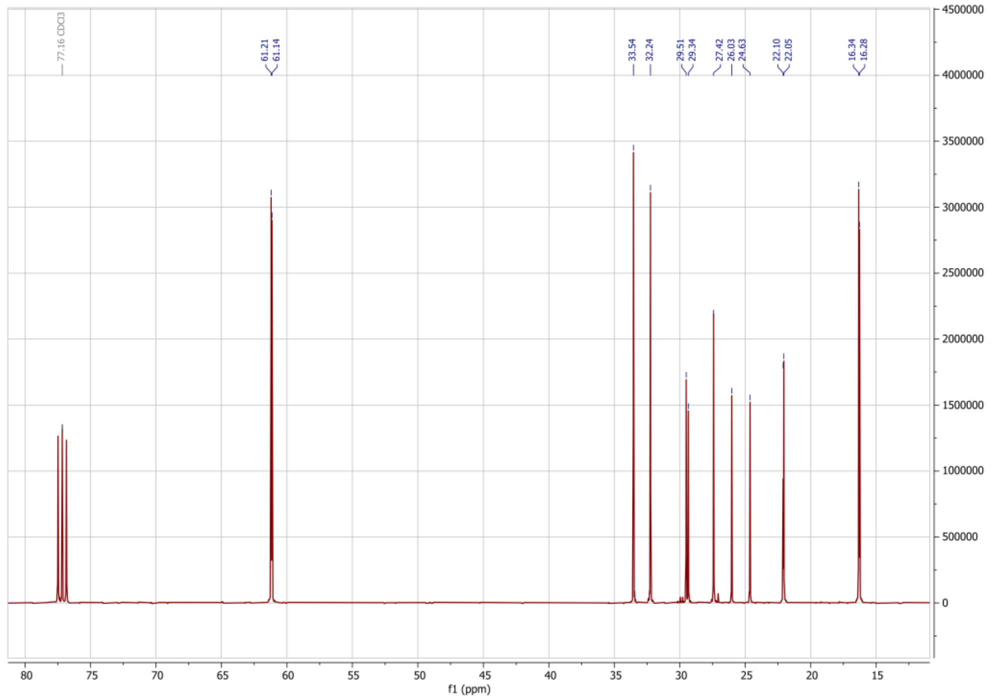

Figure S2: ^{13}C NMR spectrum of diethyl(6-bromohexyl) phosphonate.

^{13}C NMR δ ($CDCl_3$): 61.18 d (O-CH_2), 33.54 (CH_2-Br), 32.24 (CH_2-CH_2), 29.43 d, 27.42, 25.33 d (CH_2-P), 22.08 (CH_2-CH_2), 16.31 (O-CH_2-CH_3) ppm.

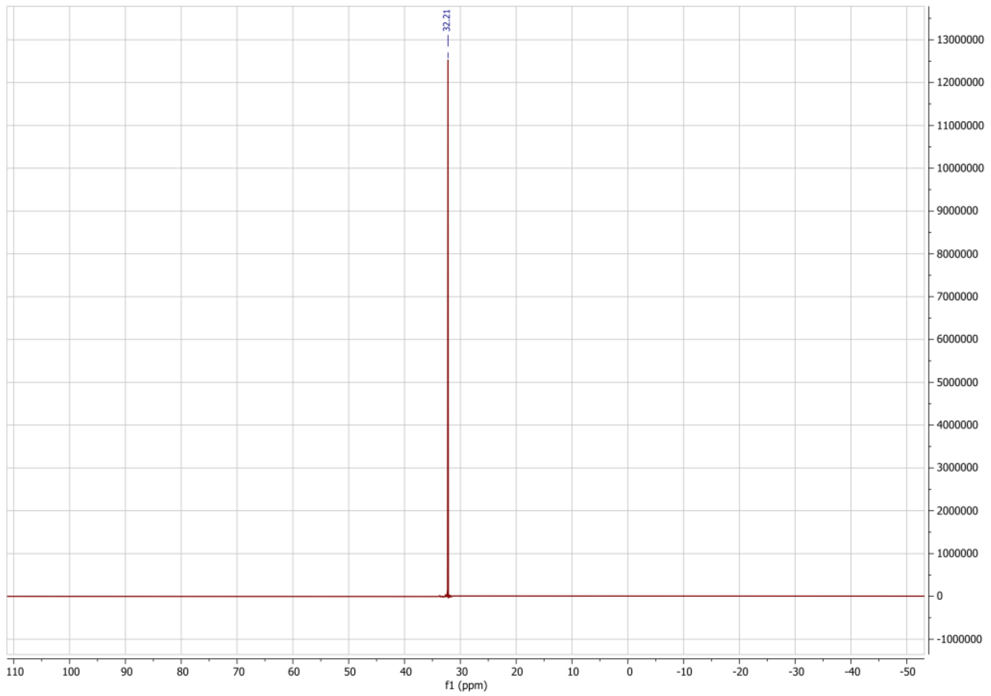

Figure S3: ^{31}P NMR spectrum of diethyl(6-bromohexyl) phosphonate.

^{31}P NMR δ ($CDCl_3$): 32.21 ppm

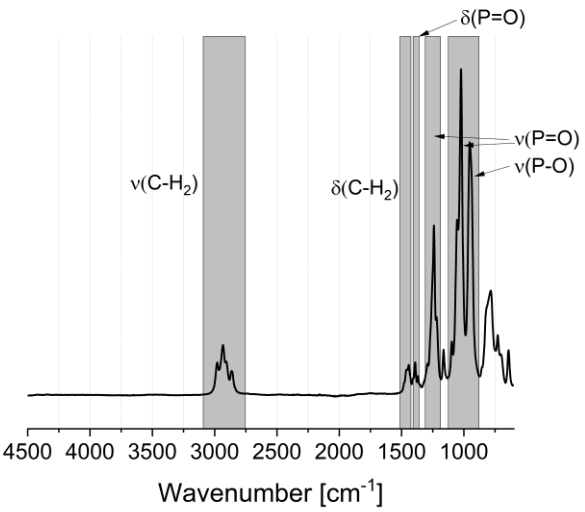

Figure S4: FTIR spectrum of diethyl(6-bromohexyl) phosphonate.

FTIR: 2982 (ν_{as} C-H_2), 2933 (ν_s C-H_2), 2864 (ν_s C-H_2), 1439 (δ C-H_2), 1394, 1242 (ν P=O), 1167, 1101, 1024, 955 (ν P-OH), 787, 644 cm^{-1}.

6-(Diethoxyphosphoryl)-N,N,N-trimethylhexan-1-ammonium bromide: 4.20 g (14 mmol) of diethyl-(6-bromohexyl) phosphonate and 10 mL (42 mmol) of trimethylamine (33% in ethanol) was stirred for 24 hours at room temperature. The crude product was filtered, and the filtrate was freed from the solvent with a rotary evaporator.

Yield: 3.82 g, 11 mmol, 76%, white solid.

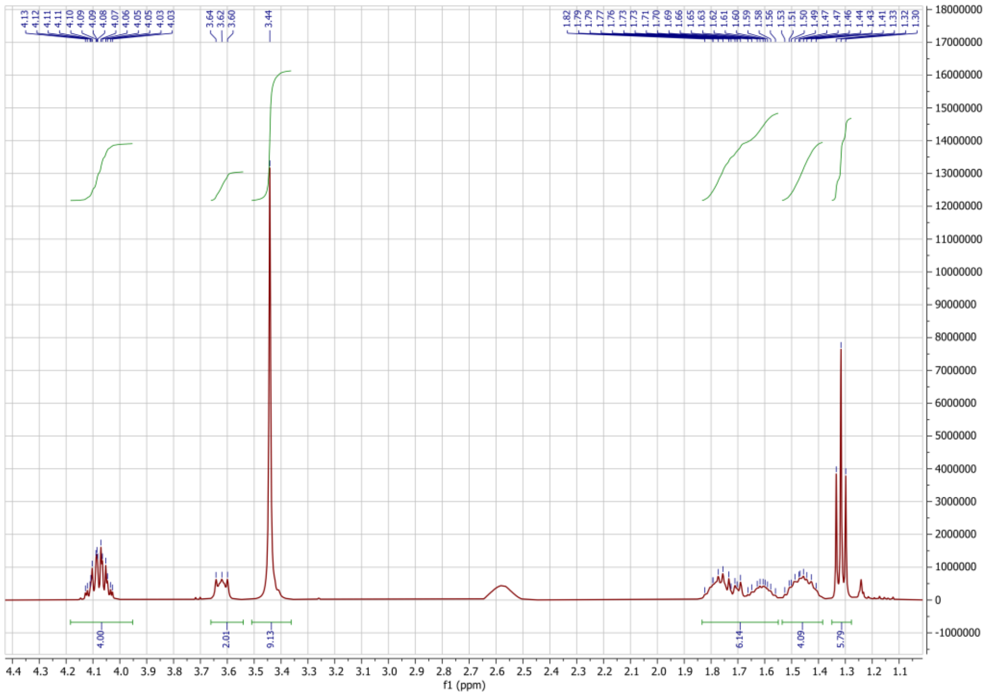

Figure S5: ^{1}H NMR spectrum of 6-(diethoxyphosphoryl)-*N*,*N*,*N*-trimethylhexan-1-ammonium bromide.

^{1}H NMR δ ($CDCl_3$) = 4.03-4.13 m (4H, O-CH_2-CH_3), 3.60-3.64 m (2H, CH_2-Br), 3.44 s (9H, CH_3), 1.41-1.82 m (10H, CH_2), 1.30-1.33 t (6H, O-CH_2) ppm.

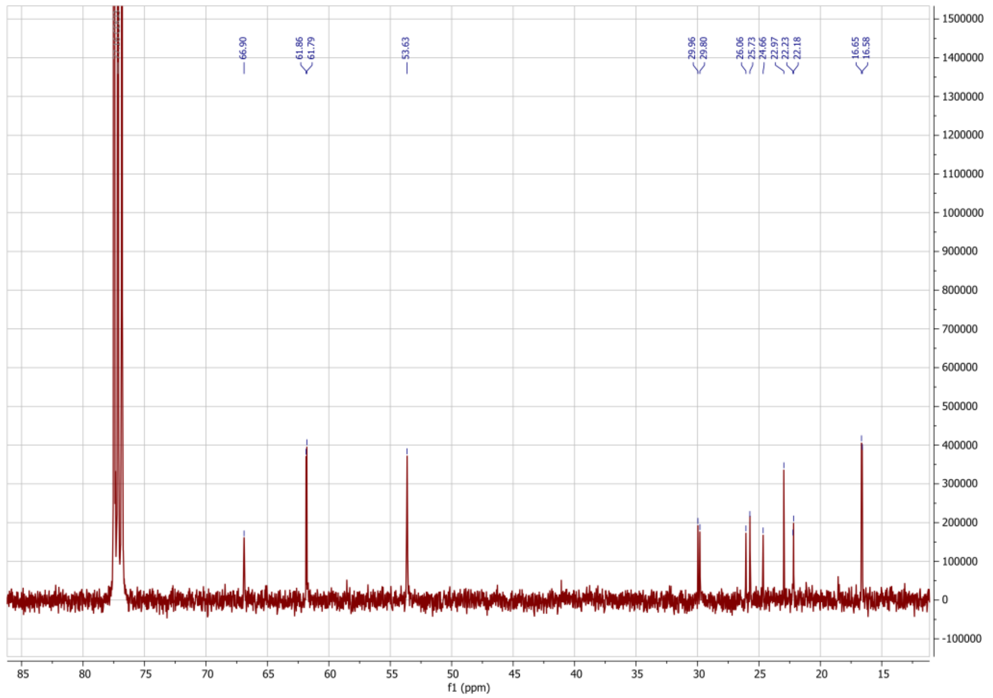

Figure S6: ^{13}C NMR spectrum of 6-(diethoxyphosphoryl)-*N*,*N*,*N*-trimethylhexan-1-ammonium bromide.

^{13}C NMR δ ($CDCl_3$): 66.90 (N-CH_3, 61.82 d (O-CH_2), 53.63 (N-CH_3), 29.83 d (CH_2-P), 25.90, 23.82 d (CH_2P), 22.23, 22.18 (CH_2-CH_2), 16.62 (O-CH_2-CH_3) ppm.

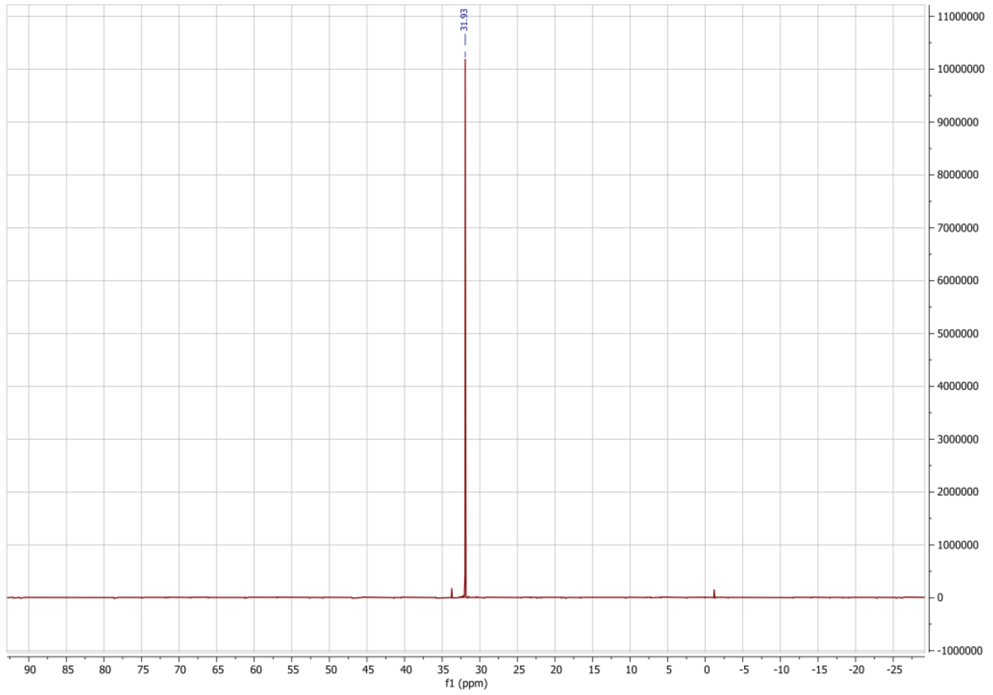

Figure S7: ^{31}P NMR spectrum of 6-(diethoxyphosphoryl)-*N*,*N*,*N*-trimethylhexan-1-ammonium bromide.

^{31}P NMR δ ($CDCl_3$): 31.93 ppm.

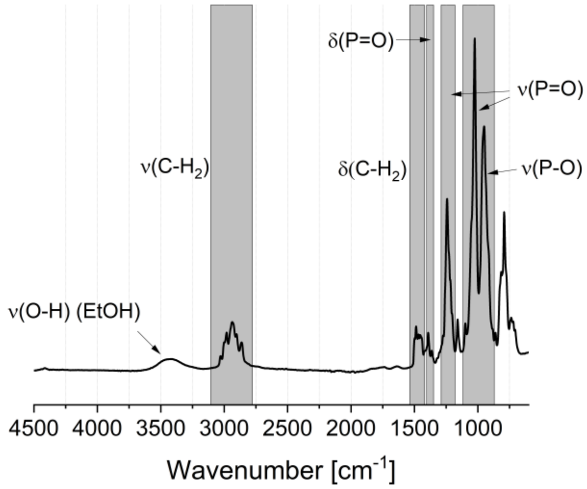

Figure S8: FTIR spectrum of 6-(diethoxyphosphoryl)-*N,N,N*-trimethylhexan-1-ammonium bromide.

FTIR: 2983 (ν_{as} C-H_2), 2941 (ν_{as} C-H_2), 2858 (ν_s C-H_2), 1488, 1394 (δ C-H_2), 1389, 1246 (ν P=O), 1163, 1095, 1026, 953 (ν P-OH), 792, 521 cm^{-1}.

N,N,N-Trimethyl-6-phosphonhexan-1-ammonium bromide: 2.35 g (6 mmol) of 6-(diethoxyphosphoryl)-*N,N,N*-trimethylhexan-1-ammonium bromide was dissolved in 10 mL dry dichloromethane at room temperature under an argon atmosphere. Then, 1.5 mL (12 mmol) of bromotrimethylsilane was added dropwise over an hour. The mixture was stirred at room temperature for a further 24 hours until the conversion to the silyl ester was completed (verified by ^{1}H NMR spectroscopy). The solvent was then removed. Subsequently, 10 mL of a methanol:water mixture (3:2) was added, and the solution was stirred at room temperature for another 24 hours. The methanol was removed with a rotary evaporator, and the product was dried in vacuo. The resulting solid was recrystallized from 2-propanol.

Yield: 1.60 g, 5 mmol, 88%, white solid.

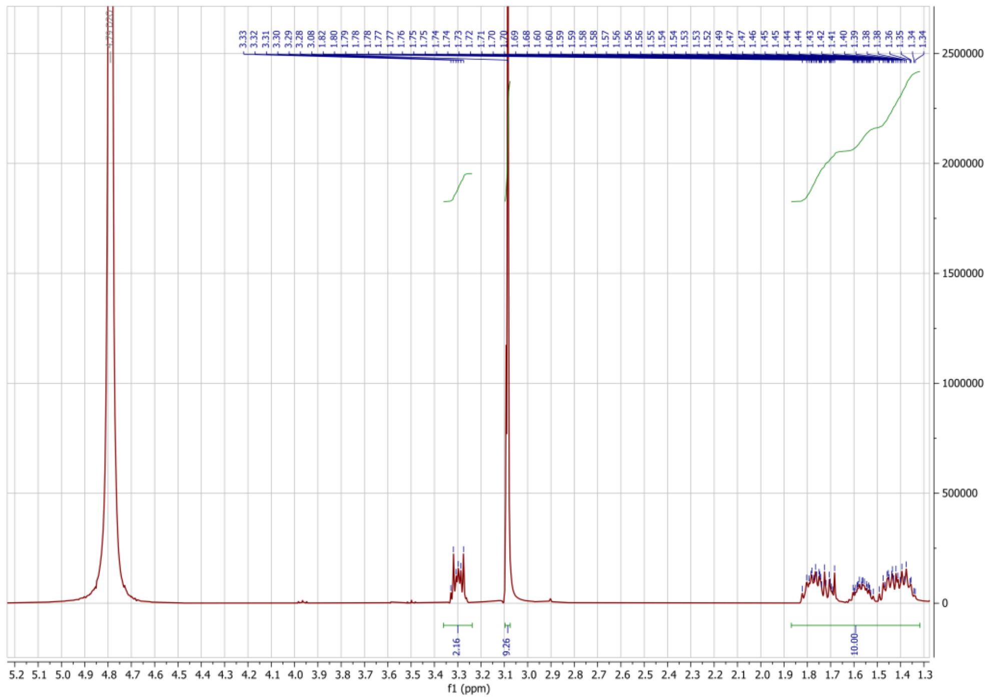

Figure S9: ^{1}H NMR spectrum of *N*,*N*,*N*-trimethyl-6-phosphonhexan-1-ammonium bromide.

^{1}H NMR δ (D_2O) = 3.28-3.33 m (2H, N-CH_2), 3.08 s (9H, N(CH_3)$_3$), 1.34-1.82 m (10H, CH_2) ppm.

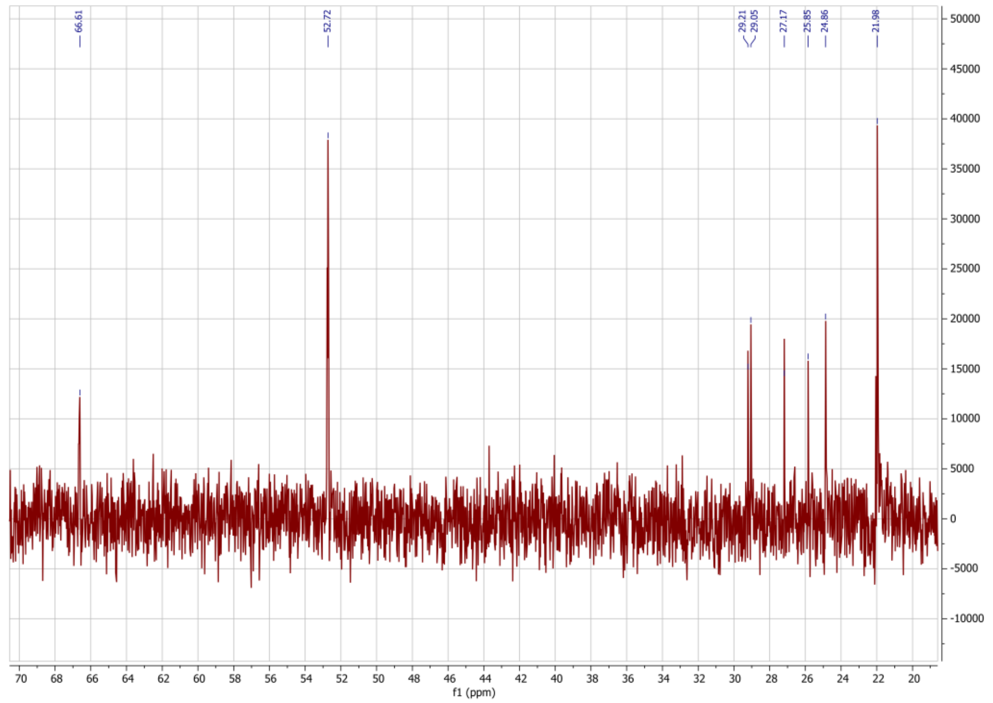

Figure S10: ^{13}C NMR spectrum of *N*,*N*,*N*-trimethyl-6-phosphonhexan-1-ammonium bromide.

^{13}C NMR δ (D_2O): 66.61 (N-CH_2), 52.72 (N-CH_3), 29.13 d (CH_2-P), 27.17, 25.85, 24.86, 21.98 (CH_2-CH_2) ppm.

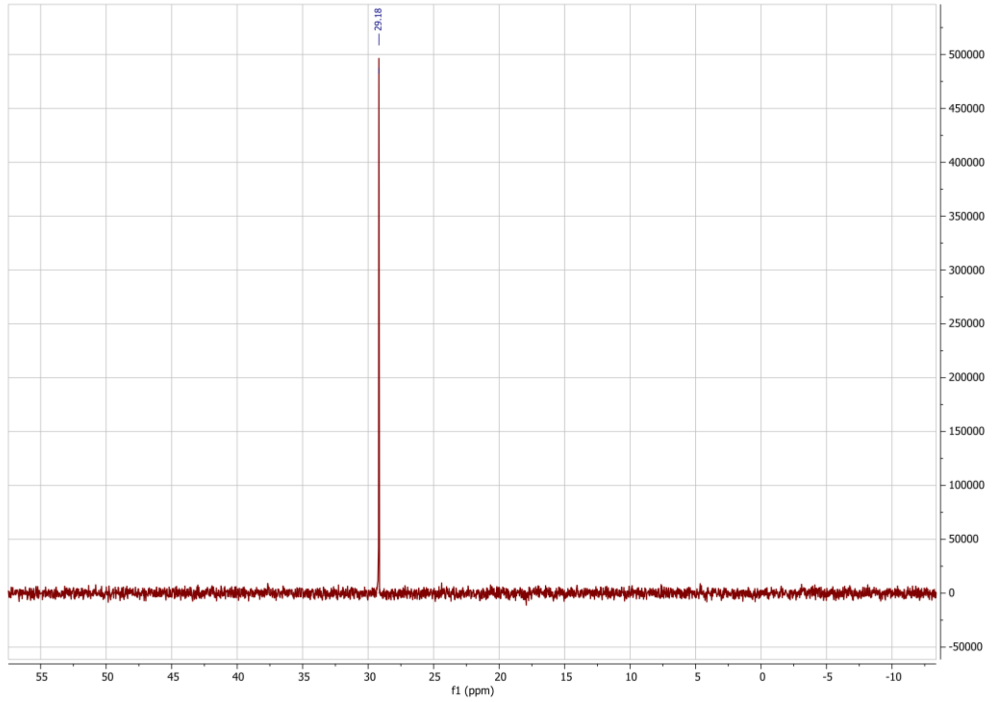

Figure S11: ^{31}P NMR spectrum of *N*,*N*,*N*-trimethyl-6-phosphonhexan-1-ammonium bromide.

^{31}P NMR δ (D_2O): 30.04 ppm.

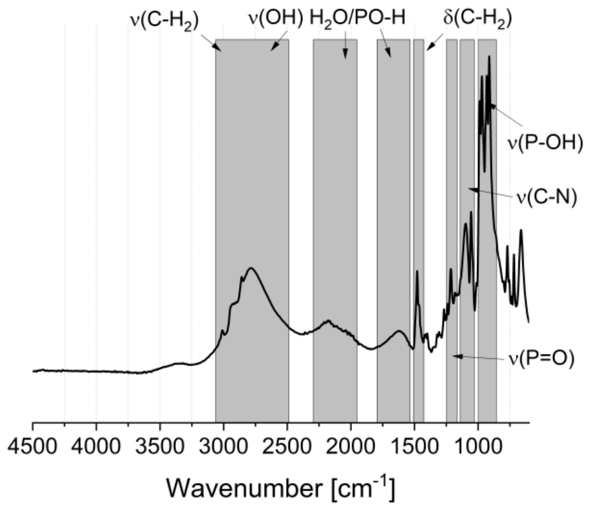

Figure S12: FTIR spectrum of *N*,*N*,*N*-trimethyl-6-phosphonhexan-1-ammonium bromide.

FTIR: 2943 (ν_{as} C-H_2), 2933 (ν_{as} C-H_2), 2858 (ν_s C-H_2), 2156, 1637 (H_2O) 1481 (δ C-H_2), 1217 (ν P=O), 1105, 1055 (ν C-N), 989, 970, 933 (ν P-OH), 914, 768, 717, 660 cm^{-1}.

CHN_{theo} ($C_9H_{23}BrNO_3P$): C: 35.54; H: 7.62; N: 4.61, CHN_{exp}: C: 33.90, H: 7,07, N: 4,48.

2. Synthesis and characterization of OA@Fe_XO_Y and $^{0.200}P$@Fe_XO_Y.

The synthesis was performed according to a previously described procedure.[2] The particles were synthesized by thermal decomposition of Fe(acac)$_3$. Therefore, 3.53 g (10 mmol) Fe(acac)$_3$, 10.12 g (10 mmol) dodecane diol, 10 mL oleic acid, and 10 mL oleyl amine were

dissolved in 100 mL benzyl ether. The reaction mixture was heated to 200 °C for 30 minutes, followed by a second heating step at 300 °C for another 30 minutes. After cooling to room temperature, the particles were decanted magnetically and washed several times with ethanol (4 x 100 mL). The obtained black solid was then redispersed in 96 mL ethanol for storage. An aliquot of 2 mL was withdrawn, dried under vacuum, and weighed to estimate the concentration and reaction yield (19.0 mg / 2 mL ≙ 912 mg total mass, TGA: 78.04% after the N_2 segment).

Yield: 711 mg, 3.1 mmol, 92%, black solid.

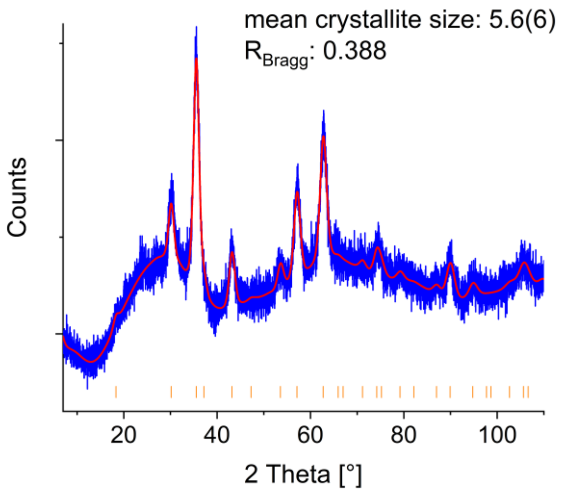

Figure S13: XRD of OA@Fe_XO_Y.

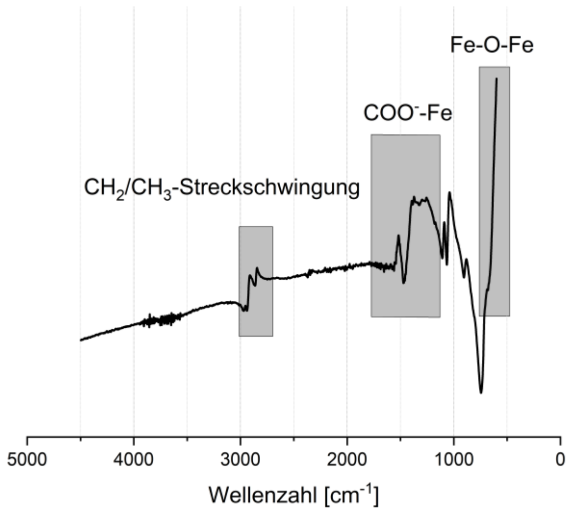

Figure S14: FTIR spectrum of OA@Fe_XO_Y.

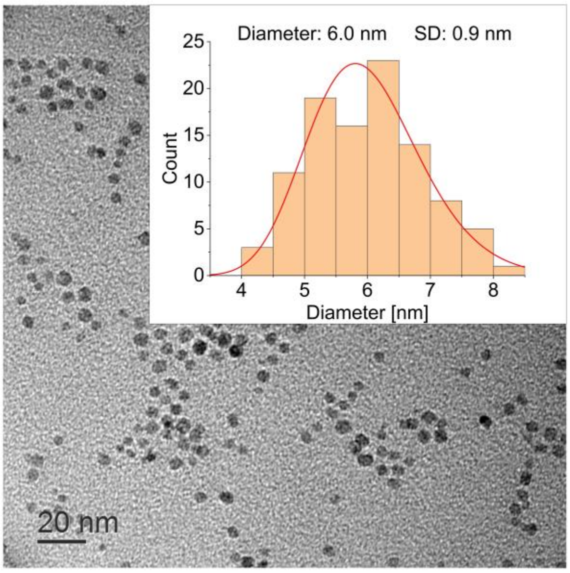

Figure S15: Transmission electron micrographs and particle size distributions of OA@Fe_XO_Y.

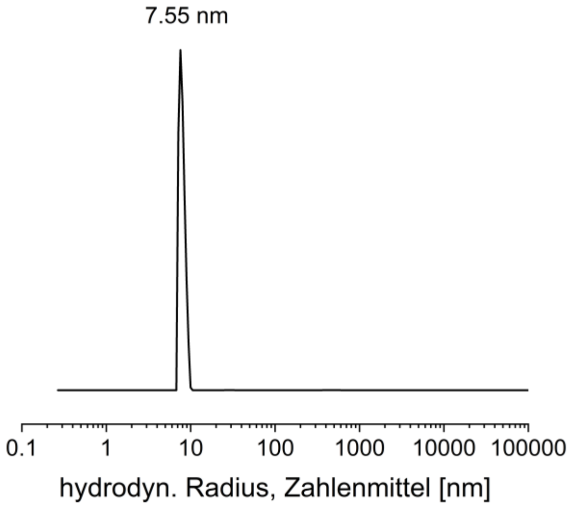

Figure S16: DLS curves of OA@Fe_XO_Y in *n*-hexane.

Ligand exchange protocol: To the oleic acid functionalized nanoparticles obtained from the thermal decomposition, 0.2 mmol per 150 mg of particles of *N*,*N*,*N*-trimethyl-6-phosphonohexane-1-ammonium bromide was added. A solvent ratio of hexane:ethanol (1:2, v:v) was chosen to ensure a stable dispersion throughout the reaction. The exchange was performed within 48 h at room temperature. The particles were then decanted magnetically. Excess phosphonic acid was removed by washing with an ethanol:hexane mixture (v:v = 1:1).

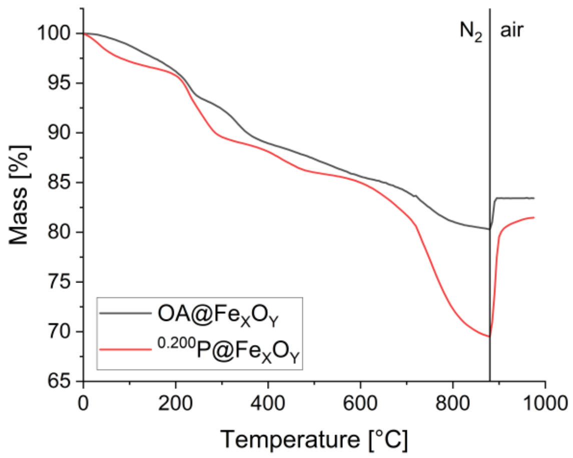

Figure S17: TGA curves of $OA@Fe_XO_Y$ and $^{0.200}P@Fe_XO_Y$.

Table S1: TGA and CHN values of oleic acid and the phosphonic acid functionalized nanoparticles.

**Sample**	**TG Residual Mass [%]**		**CHN [%]**			**Surface Coverage [mmol/g]**		
	25-880°C N_2	880-1000°C air	C	H	N	C	H	N
$OA@Fe_XO_Y$	80.29	83.42	9.64	1.80	-	0.55	0.65	-
$^{0.200}P@Fe_XO_Y$	69.55	80.16	11.30	2.48	1.00	1.50	1.54	1.03

3. Synthesis and characterization of SMBS.

The synthesis was performed according to Niederhauser.[3]

Sodium 4-(methacryloyloxy)butan-1-sulfonate: Sodium methacrylate was prepared freshly before synthesis by stirring 10.20 g (119 mmol) methacrylic acid and 9.9 g (118 mmol) sodium bicarbonate at room temperature. Completion of the reaction was determined with ^{1}H NMR spectroscopy. The obtained white solid was dried under vacuum and used without further purification. Then, 3.60 g (33 mmol) sodium methacrylate was dissolved in 35 mL ethanol and heated to reflux. Subsequently 5.00 g (37 mmol) 1,4-butane sultone was added dropwise. Heating was continued for 8 hours. After cooling to room temperature, the solvent was removed under vacuum. The obtained white solid was washed with small amounts of xylene (3x) and ethanol (3x) and dried under vacuum.

Yield: 5.43 g, 22 mmol, 67%, white solid.

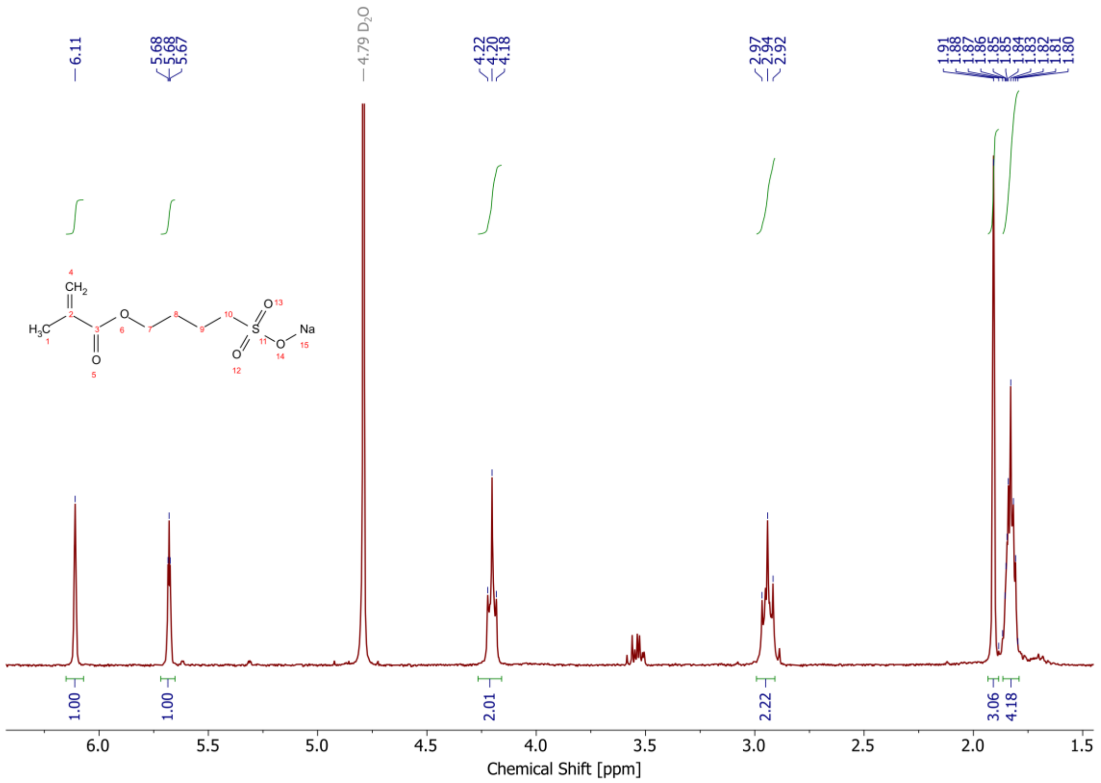

Figure S18: ^{1}H NMR spectrum of sodium 4-(methacryloyloxy)butan-1-sulfonate.

^{1}H NMR δ (D_2O) = 6.08 s (1H, =C-H), 5.63-5.67 m (1H, =C-H), 4.14-4.21 m (2H, O-CH_2), 2.88-2.95 m (2H, S-CH_2), 1.88 s (3H, CH_3), 1.78-1.82 m (4H, CH_2) ppm.

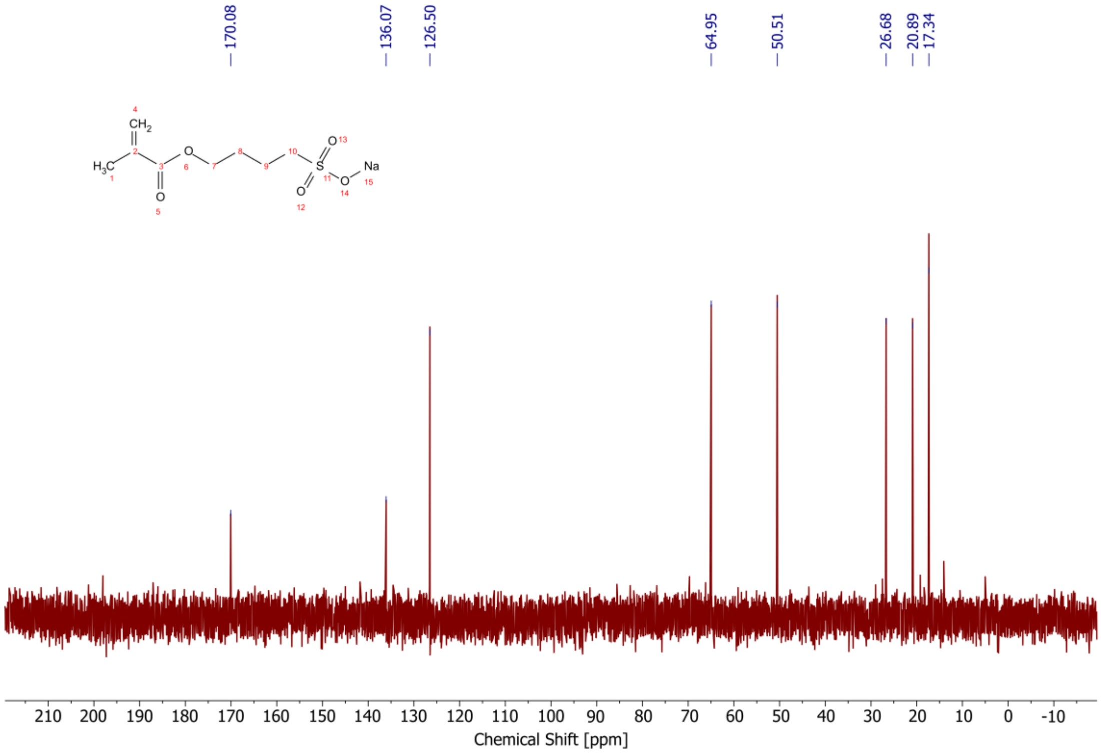

Figure S19: ^{13}C NMR spectrum of sodium 4-(methacryloyloxy)butan-1-sulfonate.

^{13}C NMR δ (D_2O): 170.06 (C=O), 136.07 (C=CH_2), 126.50 (CH_2=C), 64.95 (C-O), 50.51 (C-S), 26.68, 20.89, 17.34 (CH_2-CH_2, CH_3), ppm.

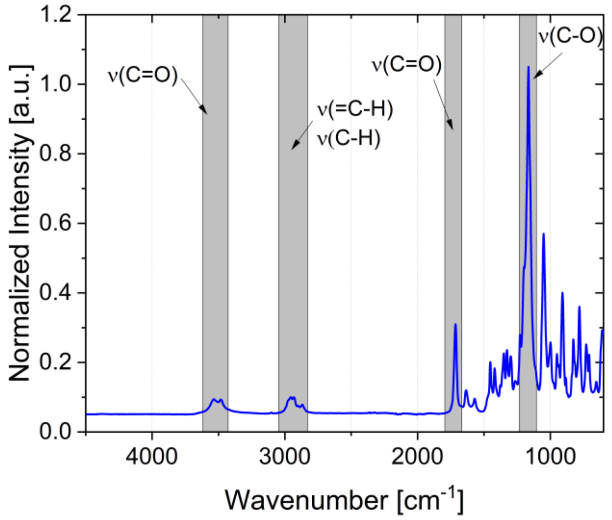

Figure S20: FTIR spectrum of sodium 4-(methacryloyloxy)butan-1-sulfonate.

FTIR: 3535 (ν_{as} C=O), 3493 (ν_{as} C=O), 2956 (ν_{as} C-H_2), 2931 (ν_{as} C-H_3), 2860 (ν_{as} C-H_3), 1716 (ν_{as} C=O), 1630, 1570 (ν_{as} C=C), 1450, 1416 (δ C=C), 1348, 1327, 1296, 1165 (ν_{as} C-O), 1049, 906 (δ =CH_2), 779 cm^{-1}.

CHN_{theo} ($C_8H_{13}NaO_5S$): C: 39.34; H: 5.37, CHN_{exp}: C: 37.24; H: 5.65.

4. Optimization of the polymerization procedure.

Polymerization was performed under standard free radical polymerization conditions. AIBN and DBPO were investigated as possible initiators. The reaction temperature and the initiator/monomer-ratio were optimized. Initially, 1 mol% AIBN was used as the initiator. Polymerization was carried out at 70 °C. A kinetic study was performed determining the optimal reaction time using pyridine as a tracer to establish the conversion. Monomer ratios of 1:3, 1:5, 1:8, and 1:10 (SMBS:DEGMA) were investigated. Samples were taken after 0.5 h, 1 h, 2 h, 3 h, 4 h, and 5 h reaction time. Figure S21 shows the proton NMR spectra.

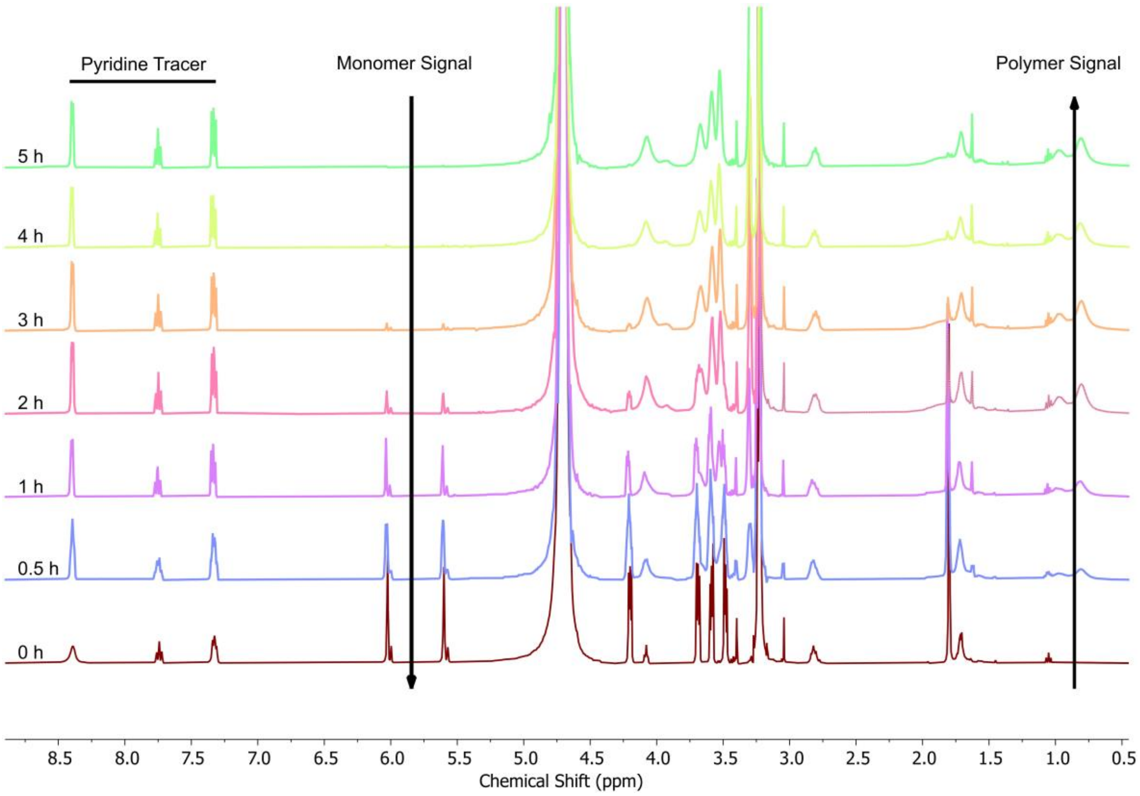

Figure S21: ^{1}H NMR of the reaction mixture of SMBS and DEGMA (1:3);
Initiator: AIBN (1 mol%); Temperature: 70 °C.

The ^{1}H NMR spectrum shows the decrease of the signal of the protons at the double bond (5.5-6.2 ppm) of the methacrylate units, indicating the conversion of the monomers. At 0.8 ppm, the signals of the polymer backbone increase. The signals were normalized to the signals of the pyridine tracer (> 7 ppm) to determine conversion. The result is shown in Figure S22.

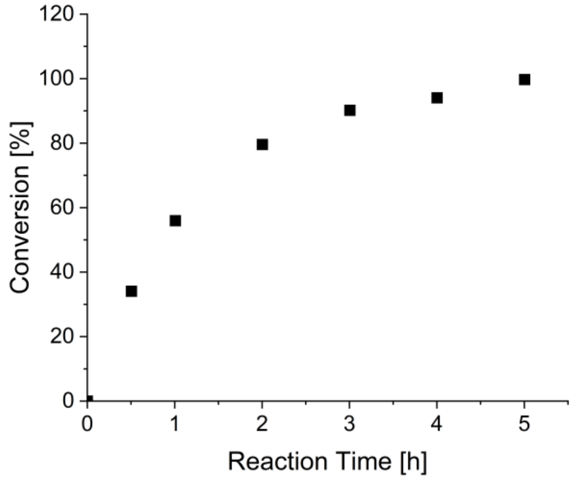

Figure S22: Conversion of SMBS and DEGMA (1:3); Initiator: AIBN (1 mol%);
Temperature: 70 °C.

The obtained conversion diagram shows the typical course for a FRP.[4,5] Except for the polymer obtained with a monomer ratio of 1:3 (SMBS:DEGMA), all systems precipitate during polymerization and are insoluble in all common solvents. Since solubility is necessary for the

composite synthesis, the initiator concentration was increased to obtain shorter polymer chain lengths. The following experiments were performed exemplarily for the 1:8 system (SMBS:DEGMA), as it showed the worst solubility. The obtained conversion dependent on the reaction time is shown in Figure S23.

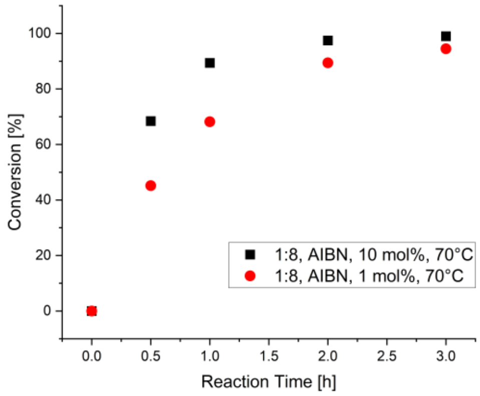

Figure S23: Comparison between the conversion of SMBS and DEGMA (1:8) at AIBN concentrations of 1 mol% (red circles) and 10 mol% (black squares); Temperature: 70 °C.

As to be expected, the reaction speed increased with the increased amount of initiator. Nonetheless, the obtained polymers were still insoluble. Due to the already extremely high initiator to monomer ratio, oligomers rather than polymers are to be expected and a further increase of the ratio seems not expedient, as chain transfer on polymers appears to occur early on and long insoluble chains are formed despite the high initiator concentration. As a result, DBPO was used as an initiator. It has lower activation rate than AIBN, even though that does not always correlate with overall reaction speeds.[4,6] First, the reaction was carried out at 85°C, which is the usual reaction temperature for DBPO. Figure S24 shows the conversion of the polymerization with a comparison of AIBN and DBPO.

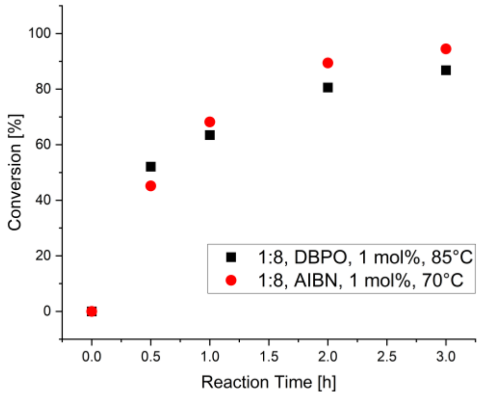

Figure S24: Comparison between the conversion of SMBS and DEGMA (1:8) using AIBN (red circles) and DBPO (black squares); Initiator concentration: 1 mol%, Temperature: 70 °C (AIBN), 85 °C (DBPO).

The polymerizations show comparable reaction speeds for the two initiators. The obtained polymers synthesized with DBPO show swelling in contact with water but are still not soluble. In a next step, the reaction temperature was considered as an optimization parameter. In order

to better control the reaction, the reaction temperature was gradually lowered from 85 °C to 70 °C and then to 60 °C. The amount of initiator was fixed at 1 mol% (Figure S25).

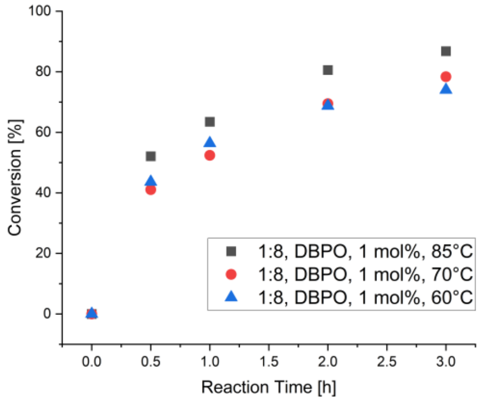

Figure S25: Comparison between the conversion of SMBS and DEGMA (1:8); Initiator: DBPO (1 mol%), Temperature: 85 °C (black squares), 70 °C (red circles) and 60 °C (blue triangles).

The conversion rate of the polymerization decreases noticeably from 85°C to 70°C. In contrast, no major differences between the reaction temperatures of 70 °C and 60 °C can be observed. However, the properties change significantly. The solubility of the polymers improves as the reaction temperature decreases. The 1:8 samples synthesized at 60 °C are still not completely soluble, but allow for further processing to the composites and show comparable conversion rates as at 85°C. Therefore, the further investigated polymers were synthesized at that temperature. Under the optimized reaction conditions, polymerizations with the 1:5, 1:8, and 1:10 monomer ratios (SMBS:DEGMA) were performed. The conversion is shown in Figure S26.

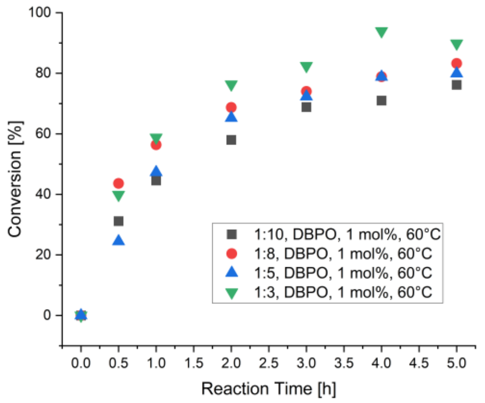

Figure S26: Comparison between the conversion of SMBS and DEGMA for the monomer compositions 1:3 (green triangles), 1:5 (blue triangles), 1:8 (red circles), and 1:10 (black squares); Initiator: DBPO (1 mol%); Temperature: 60 °C.

A reaction time of five hours was chosen. The polymerizations were carried out without the pyridine tracer under the optimized conditions.

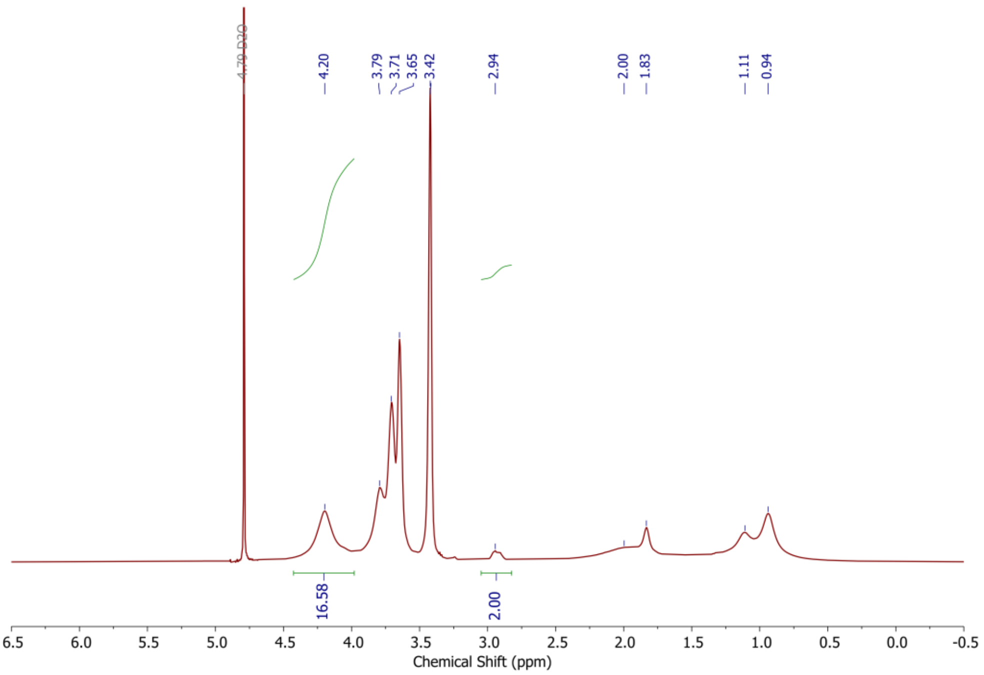

Figure S27: ^{1}H NMR of SMBS and DEGMA (1:5); Initiator: DBPO (1 mol%); Temperature: 60 °C; Reaction time: 5 h.

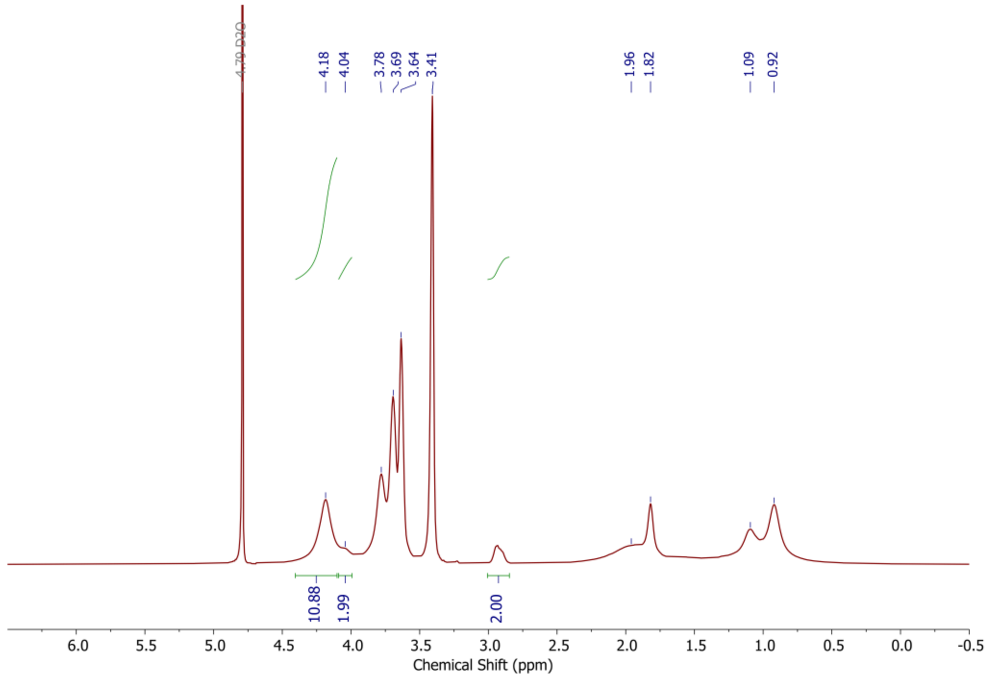

Figure S28: ^{1}H NMR of SMBS and DEGMA (1:8); Initiator: DBPO (1 mol%); Temperature: 60 °C; Reaction time: 5 h.

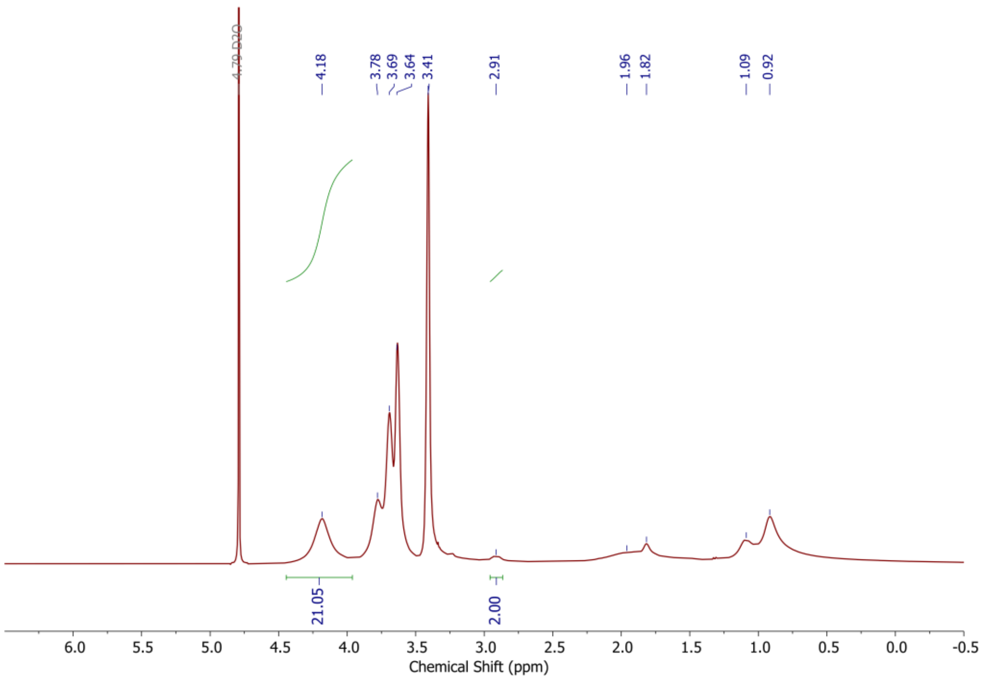

Figure S29: ^{1}H NMR of SMBS and DEGMA (1:10); Initiator: DBPO (1 mol%); Temperature: 60 °C; Reaction time: 5 h.

Table S2: CHN analysis of the synthesized polymers.

**Sample**	**CHN [%]**			
	C	H	N	S
$^{22.4}$Pol	54.32	7.55	-	0.72
$^{19.5}$Pol	54.80	7.81	-	0.82
$^{13.7}$Pol	53.44	7.52	-	1.14
$^{9.4}$Pol	54.10	7.90	-	1.59

5. Water adsorption studies of the synthesized polymers and composites

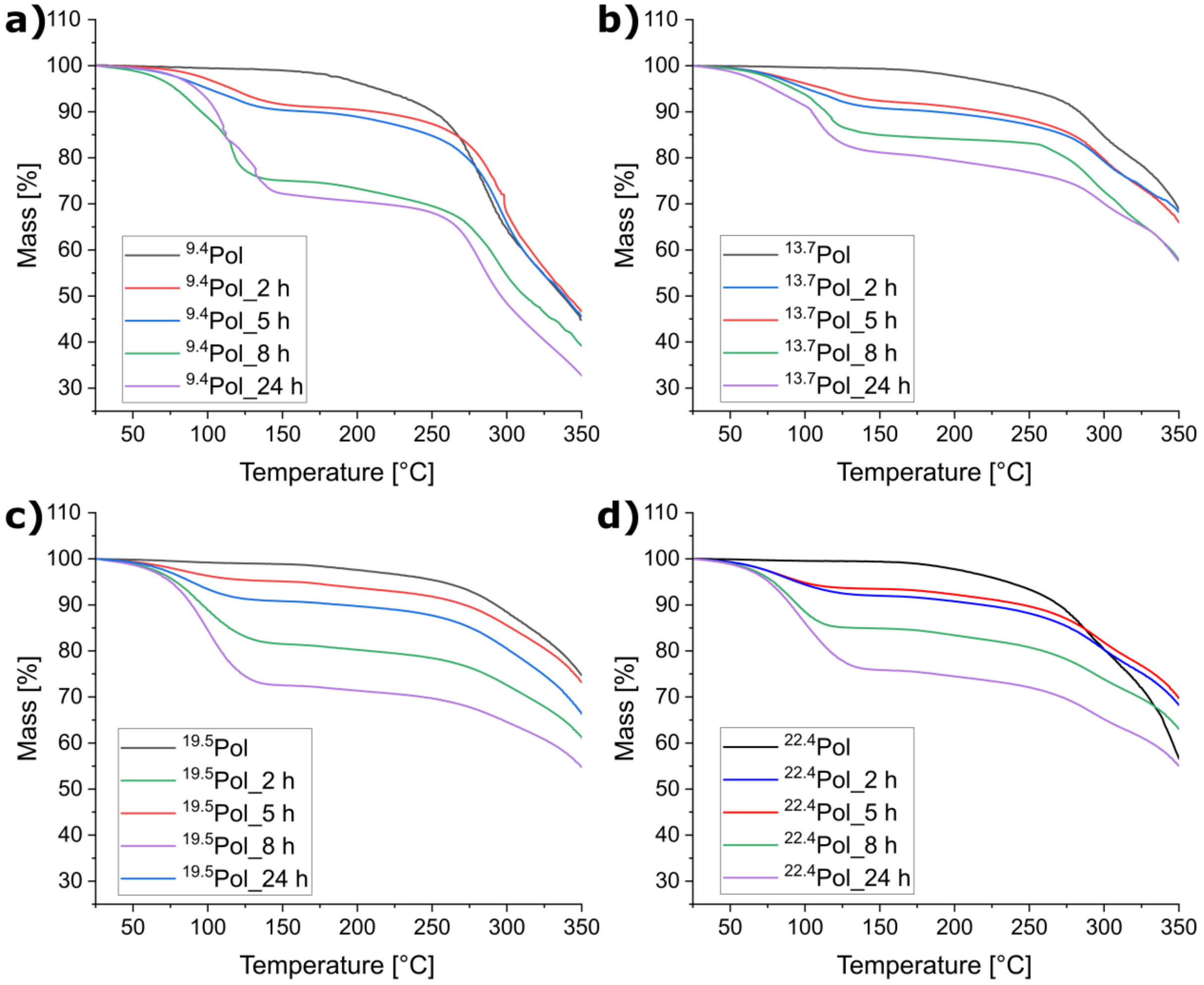

Figure S30: TGA curves of the a) $^{9.4}$Pol, b) $^{13.7}$Pol, c) $^{19.5}$Pol, and d) $^{22.4}$Pol systems after varying storage times under humid conditions.

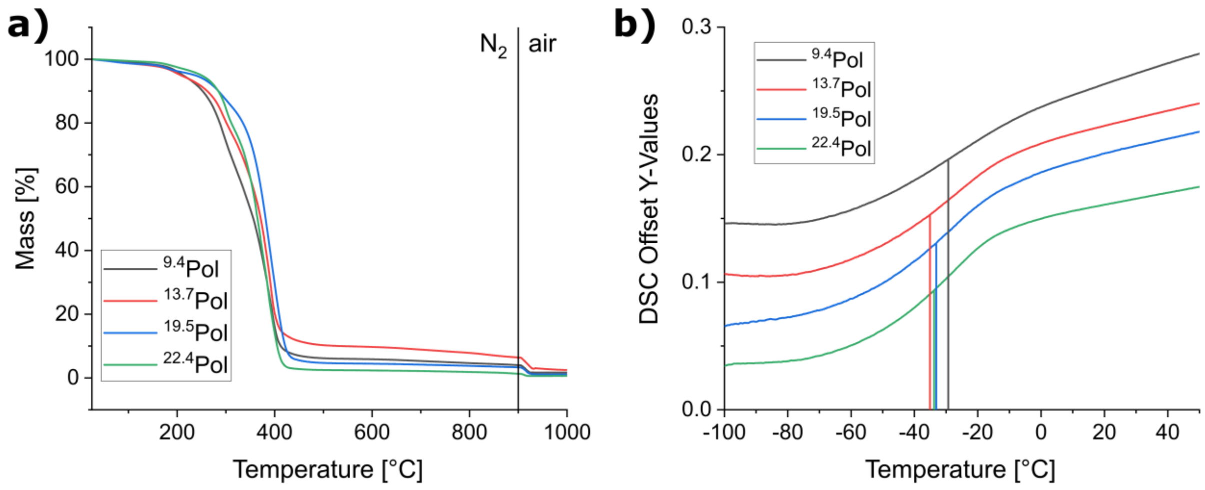

Figure S31: a) TGA and b) DSC curves of the $^{9.4}$Pol (black), $^{13.7}$Pol (red), $^{19.5}$Pol (blue), and $^{22.4}$Pol (green) polymers obtained by FRP.

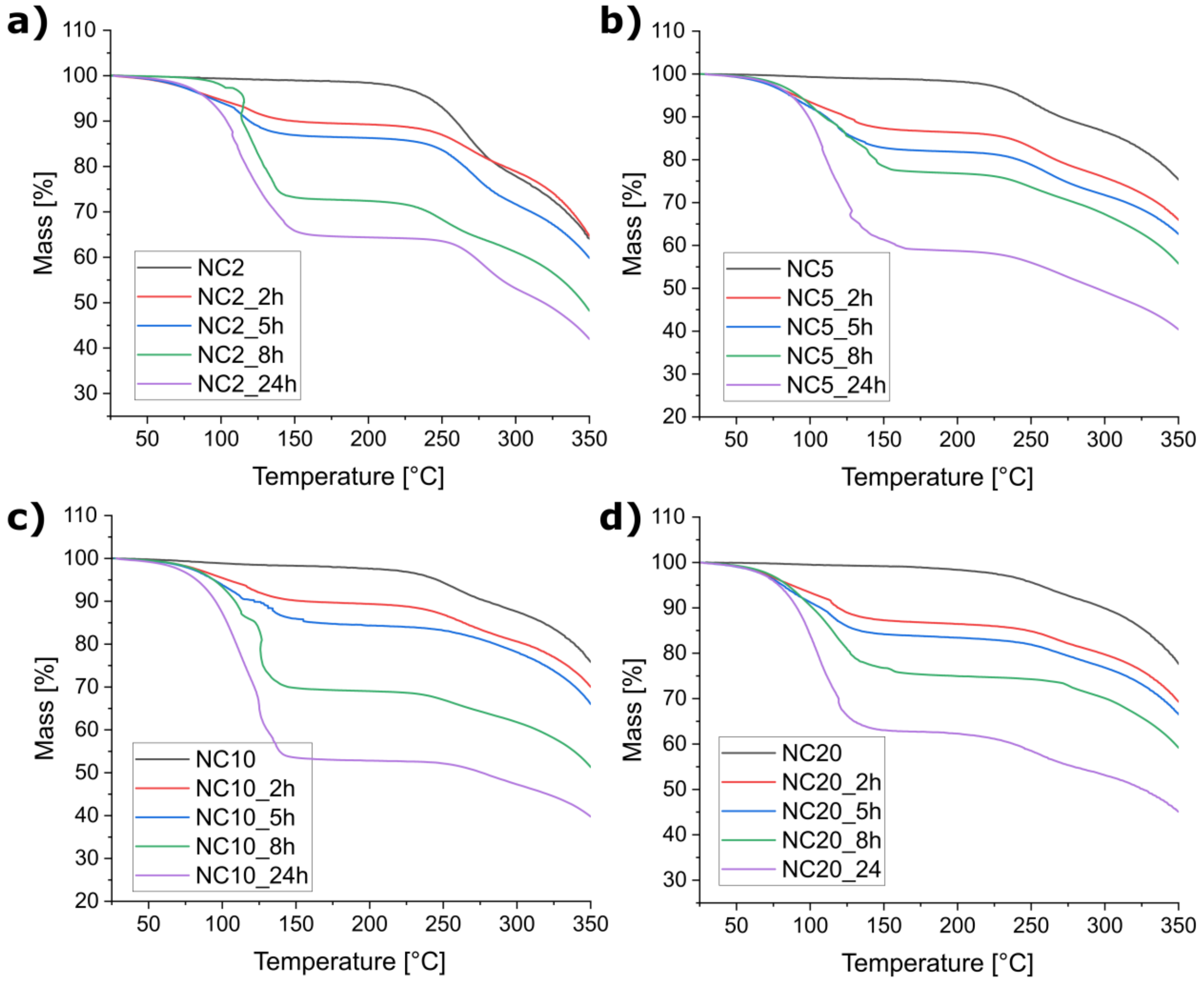

Figure S32: TGA curves for the a) NC2, b) NC5, c) NC10, and d) NC20 systems after varying storage times at high humidity.

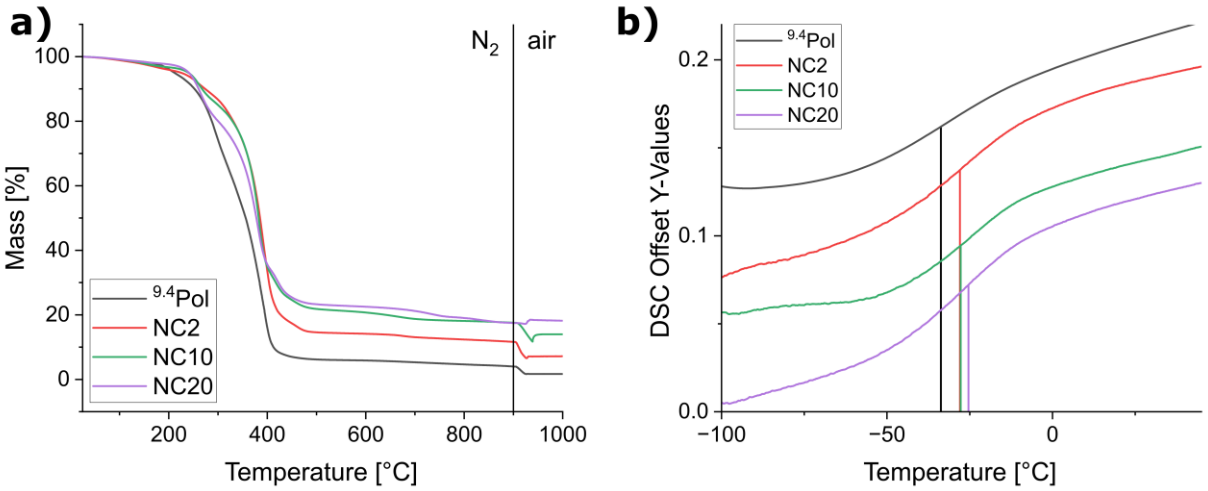

Figure S33: a) TGA and b) DSC curves of the $^{9.4}$Pol (black), NC2 (red), NC10 (green), and NC20 (purple) samples.

6. Rheology measurements of the DOW Corning OE6630 reference samples.

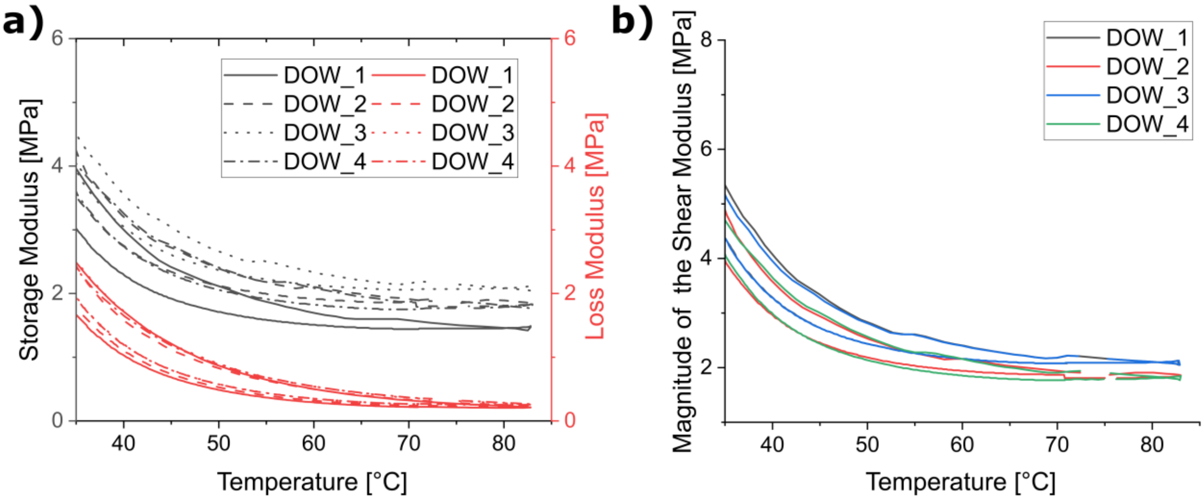

Figure S34: Reproducibility of the rheology measurements of the Dow Corning OE6630 reference samples.

7. Rheology measurements of the polymer samples.

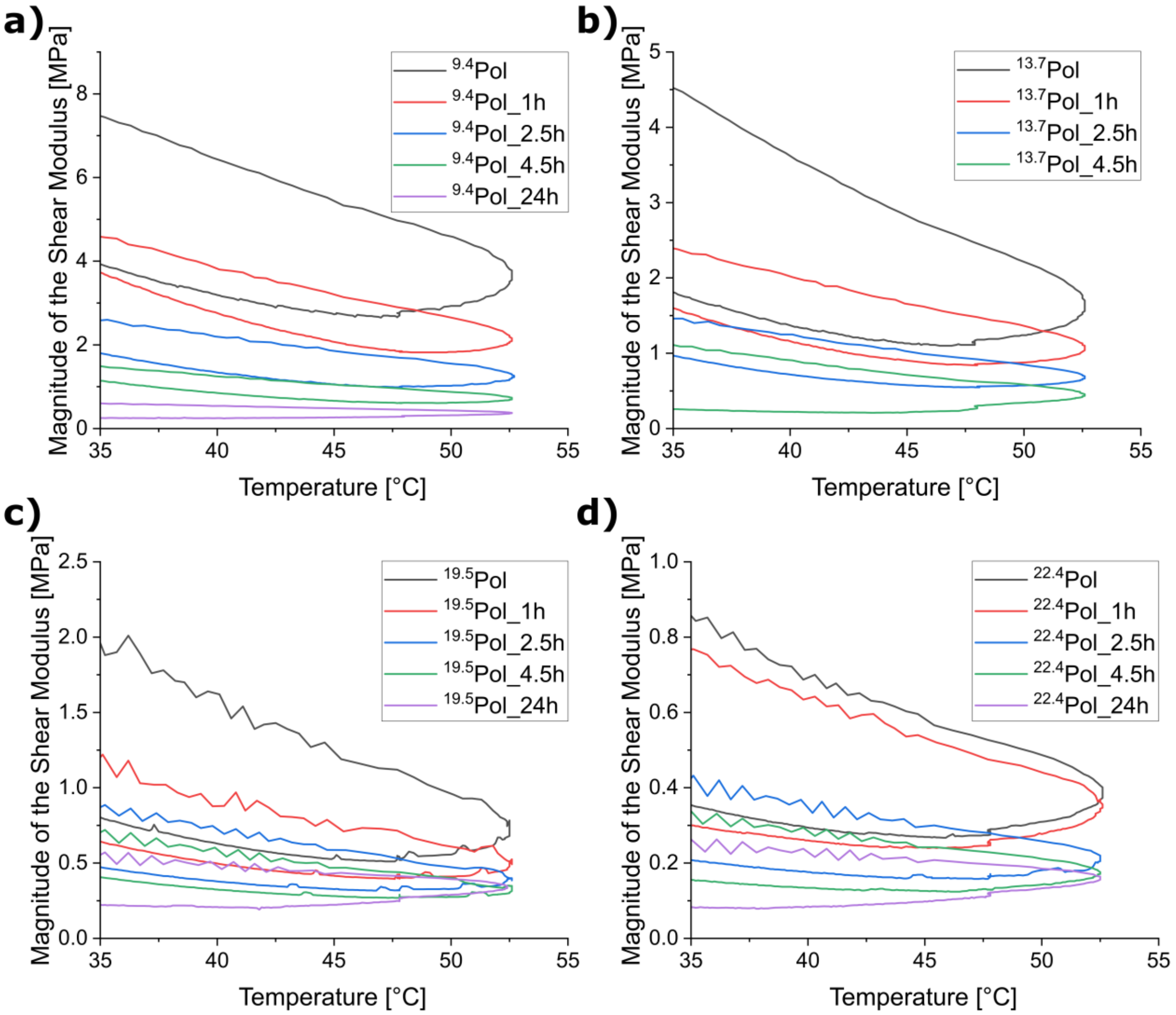

Figure S35: Magnitude of the shear modulus of the a) $^{9.4}$Pol, b) $^{13.7}$Pol, c) $^{19.5}$Pol, and d) $^{22.4}$Pol systems at different temperatures dependent on the storage time under humid conditions.

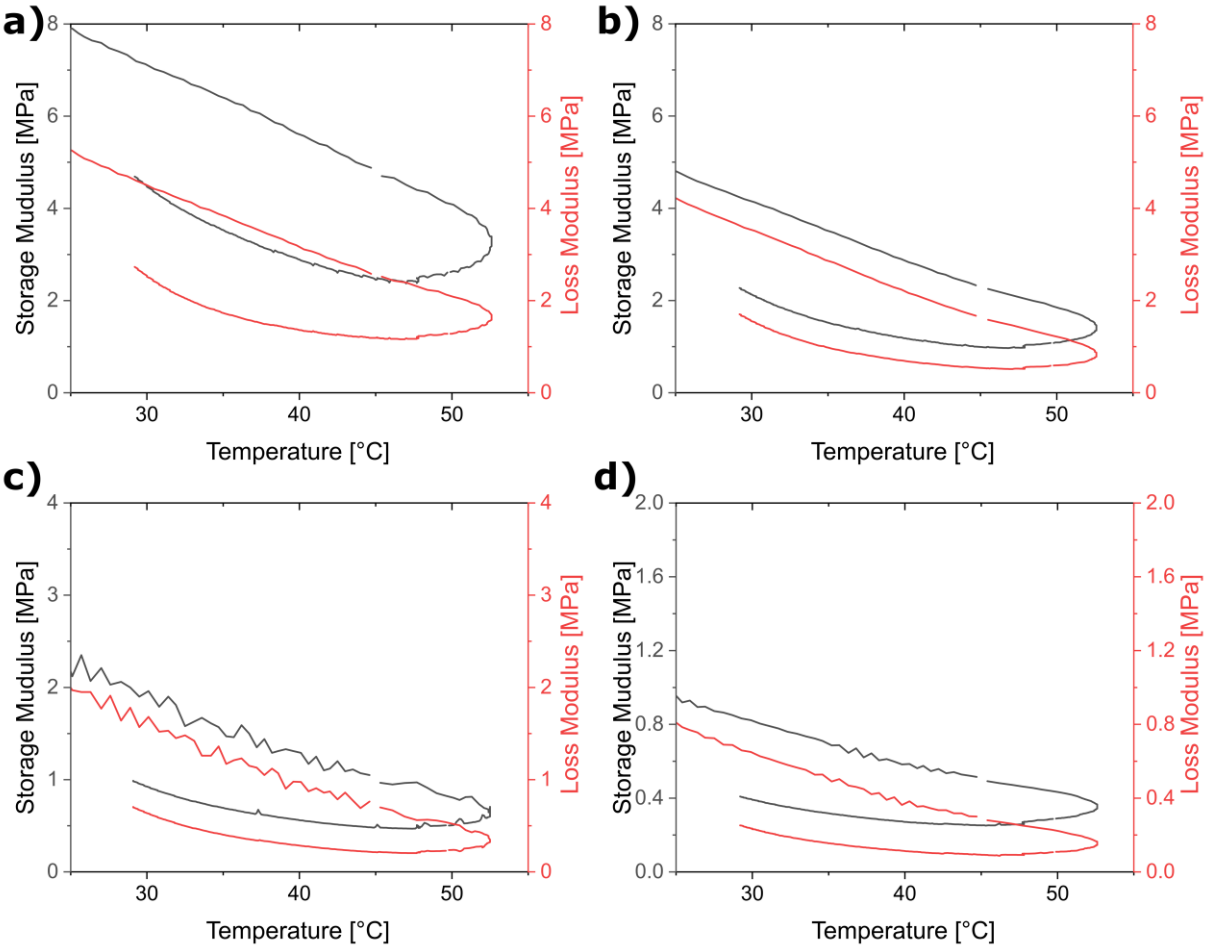

Figure S36: Storage and loss moduli for the dried polymers a) $^{9.4}$Pol, b) $^{13.7}$Pol, c) $^{19.5}$Pol, and d) $^{22.4}$Pol plotted against temperature.

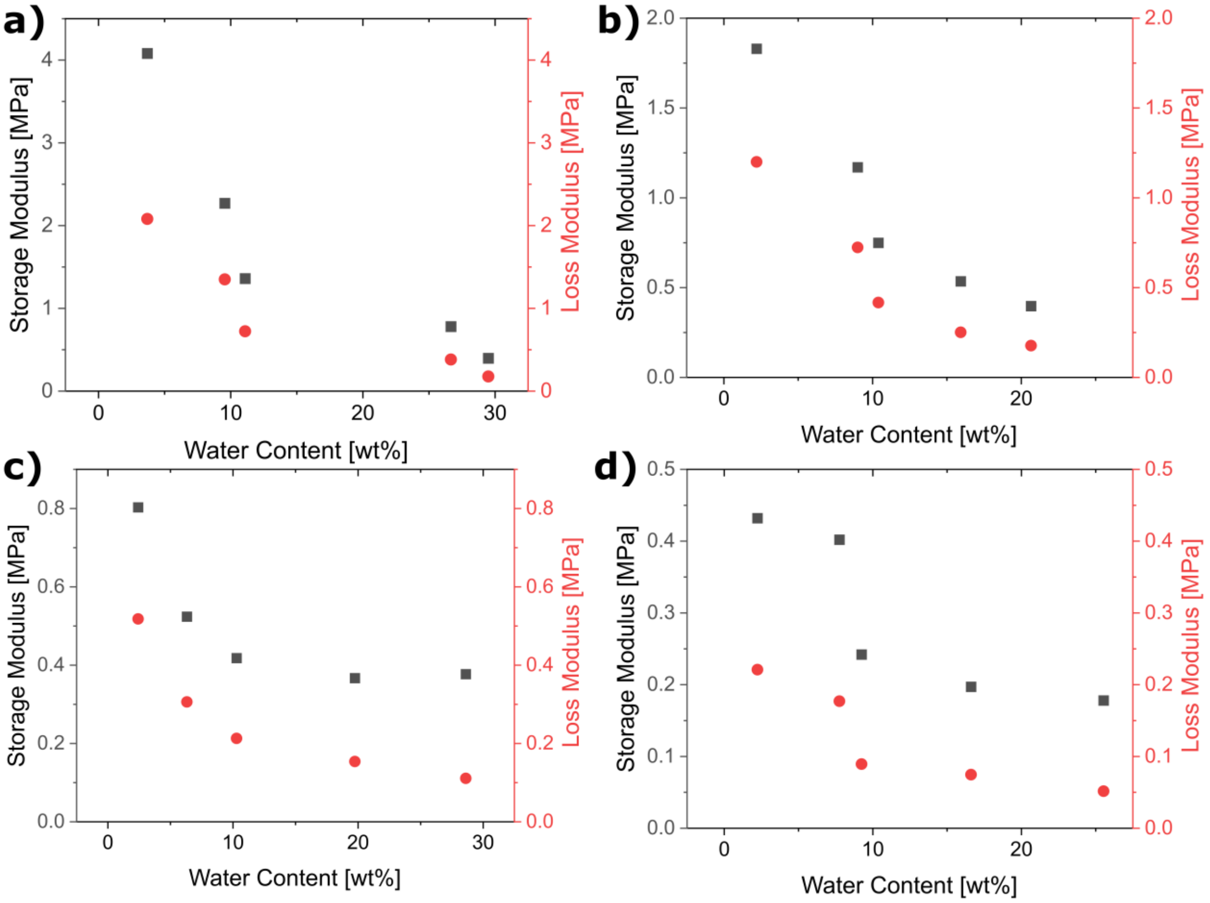

Figure S37: Storage (black squares) and loss (red circles) moduli for a) $^{9.4}$Pol, b) $^{13.7}$Pol, c) $^{19.5}$Pol, and d) $^{22.4}$Pol dependent on the water uptake upon storage at high humidity.

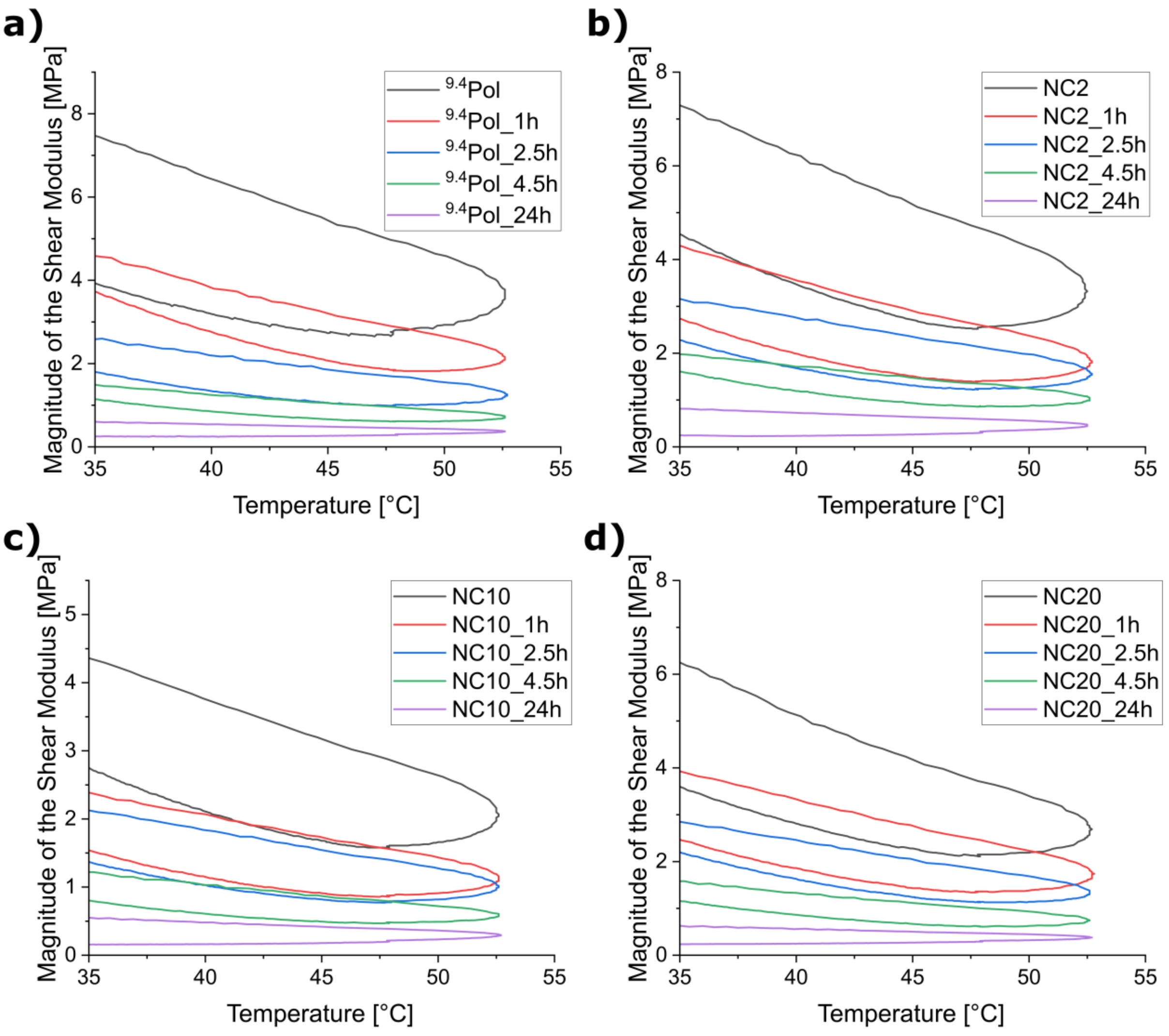

Figure S38: Magnitude of the shear modulus of the a) $^{9.4}$Pol, b) NC2, c) NC10, and d) NC20 systems at different temperatures dependent on the storage time under humid conditions.

8. TGA measurements after different drying times at 80 °C.

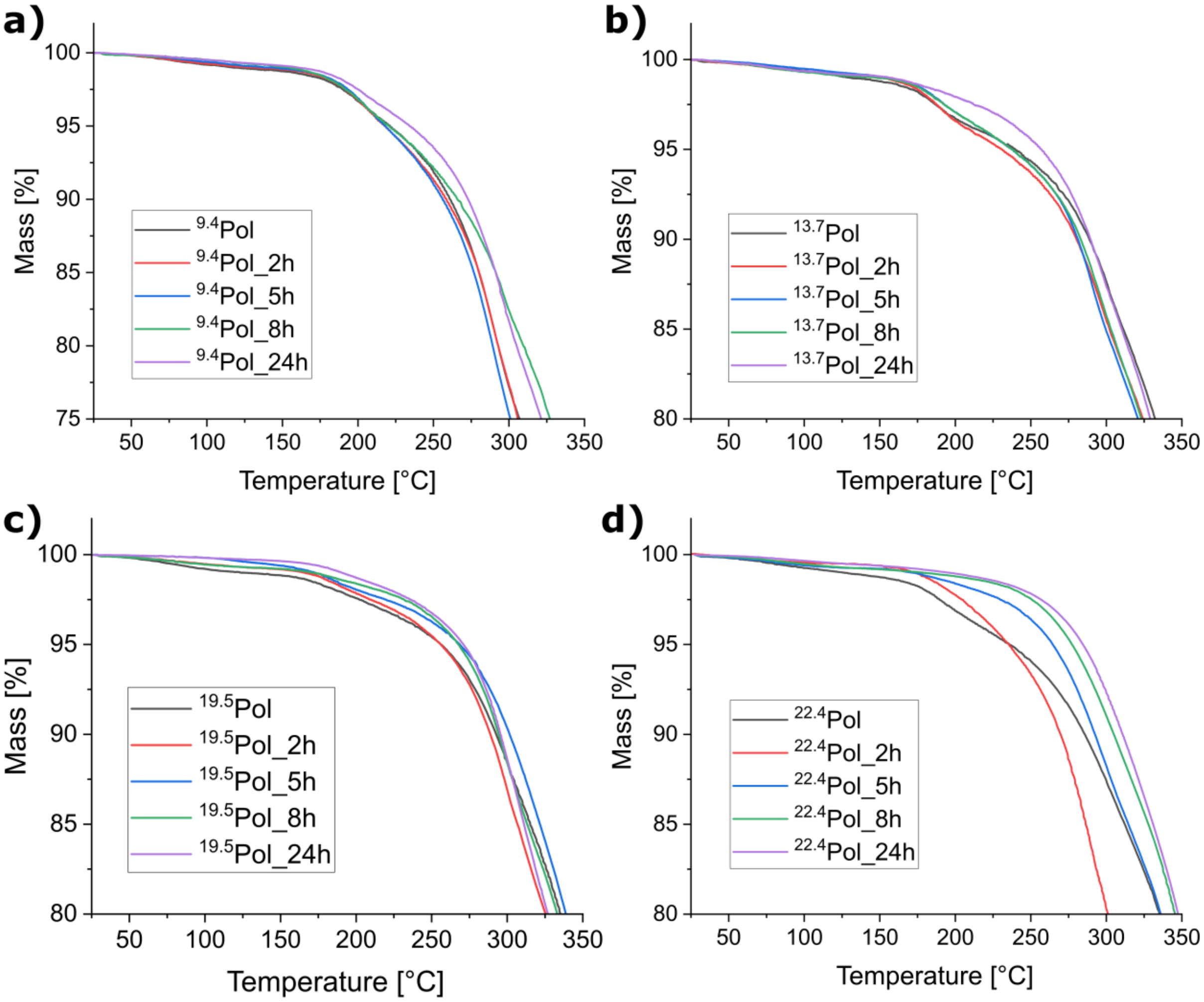

Figure S39: TGA curves of a) $^{9.4}$Pol, b) $^{13.7}$Pol, c) $^{19.5}$Pol, and d) $^{22.4}$Pol polymers dependent on the storage time at 80 °C.

9. Tensile testing of the polymer and composite samples.

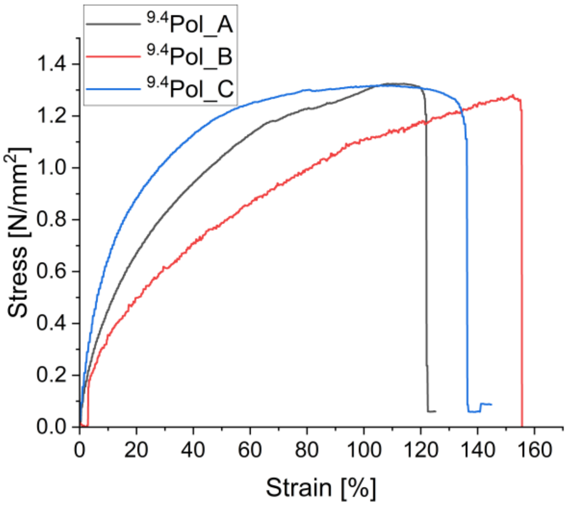

Figure S40: Stress-strain curves of the three $^{9.4}$Pol samples.

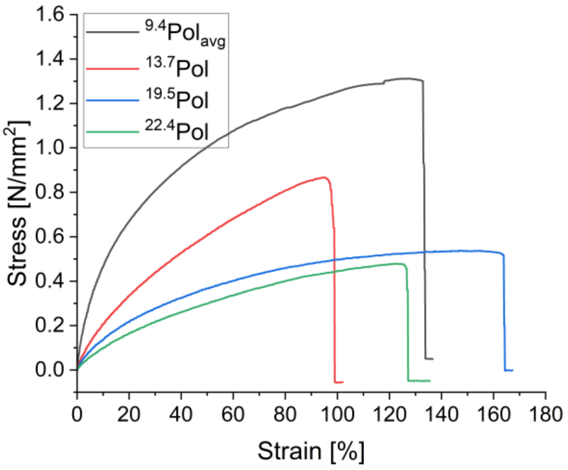

Figure S41: Stress-strain curves of the polymer samples.

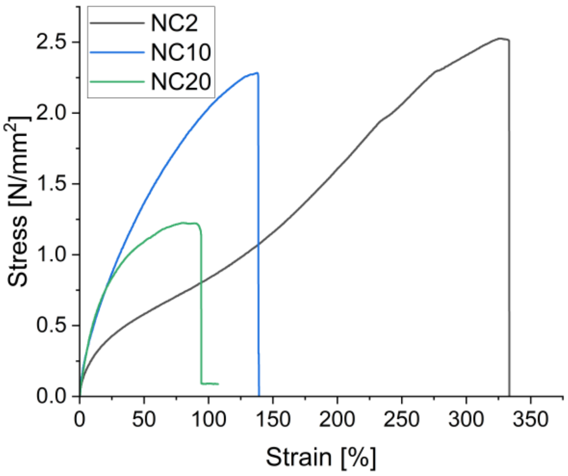

Figure S42: Stress-strain curves of the dried composite samples.

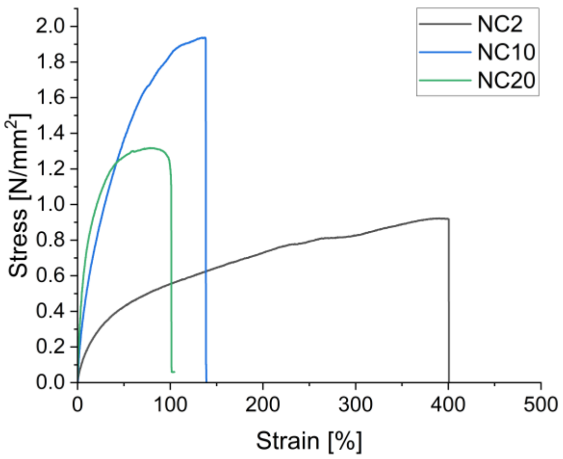

Figure S43: Stress-strain curves of the healed composite samples.

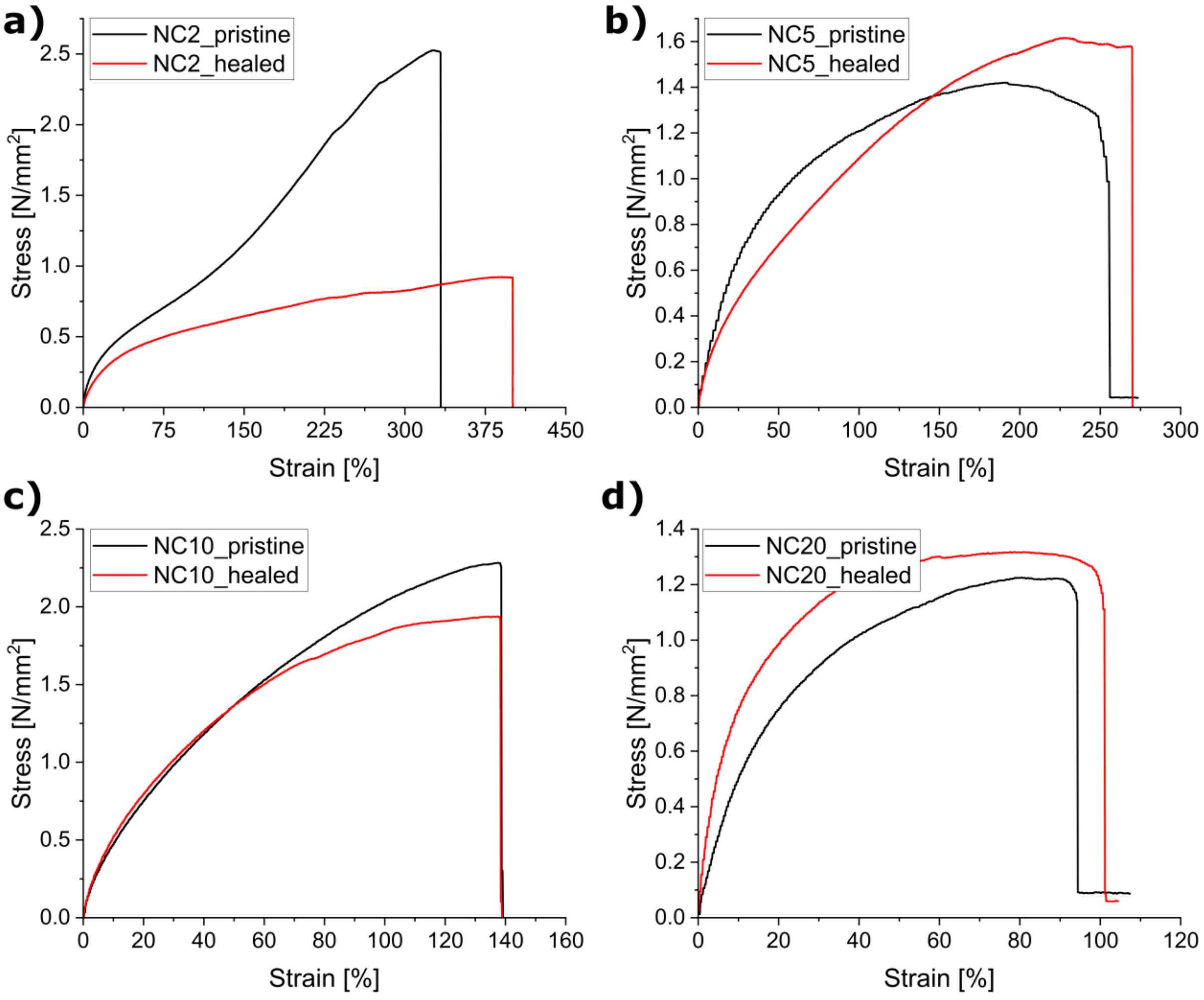

Figure S44: Stress-strain curves of the pristine composites in comparison to the healed composites.

10. Temperature Stability of the Polymer Matrix.

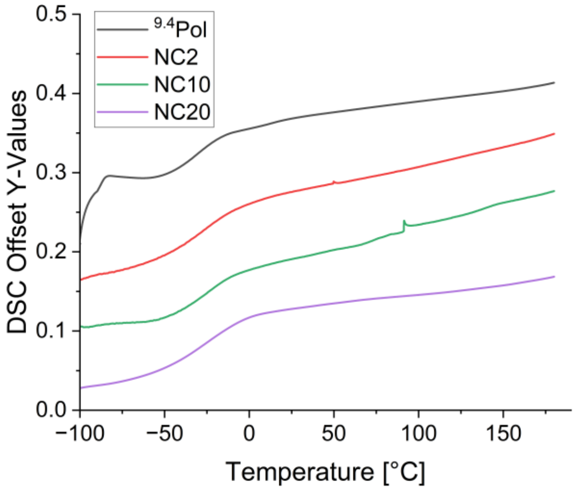

Figure S45: DSC curves of the $^{9.4}$Pol (black), NC2 (red), NC10 (green), and NC20 (purple) samples.

Since no melting signal was observed in the DSC, the stability of the crystalline areas of the polymer were investigated further exemplarily for the $^{9.4}$Pol system. For this, temperature-dependent SAXS measurements were performed. The results are shown in Figure S46.

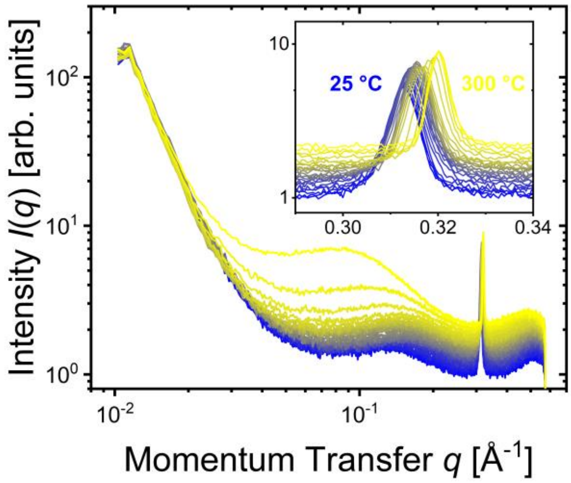

Figure S46: Temperature-dependent SAXS measurements of the $^{9.4}$Pol system from 25 °C to 300 °C in steps of 10 °C. The inset shows the scattering curve in the q-range relevant for the Bragg peak at ~0.31 Å^{-1}.

The signal at 0.3 Å^{-1} is retained even at temperatures where decomposition of the polymer begins, which is astonishing as the multiplets described for systems like poly(ethylene-co-methacrylic acid are much more temperature labile.[7] The high temperature stability of these crystalline areas explains the absence of a melting signal in DSC. It can also be observed that the signal shifts to higher q values with increasing temperature, most likely because the comprised water evaporates out.

11. Results of the SAXS Measurements.

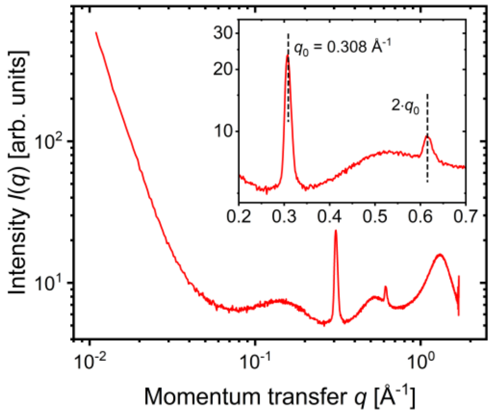

Figure S47: SAXS measurements of the $^{9.4}$Pol system.

Table S 3: Results of the SAXS measurements at 40 °C and 80 °C drying temperature.

Drying Temperature	40°C			80°C		
Parameter	R_{HS}	m	η [%]	R_{HS}	m	η [%]
NC2	10.1	2.7	32.3	10.2	3.1	33.3
NC10	9.0	3.1	26.4	9.4	3.2	24.6
NC20	7.5	2.9	25.4	7.4	3.0	25.2

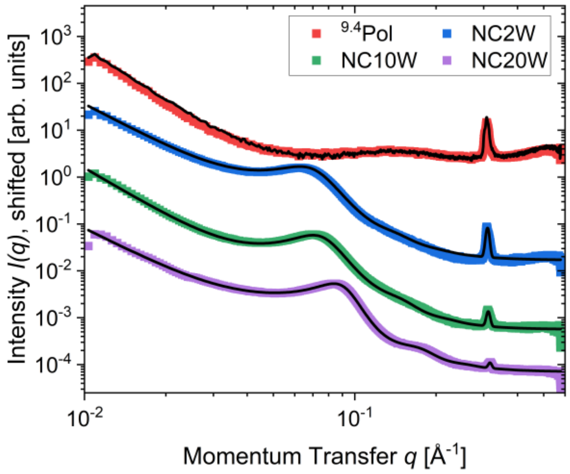

Figure S48: SAXS measurements of the composite samples under humid conditions for 2 h with particle wt% as indicated in the graph. Black lines are fitted to the data according to eq.(1).

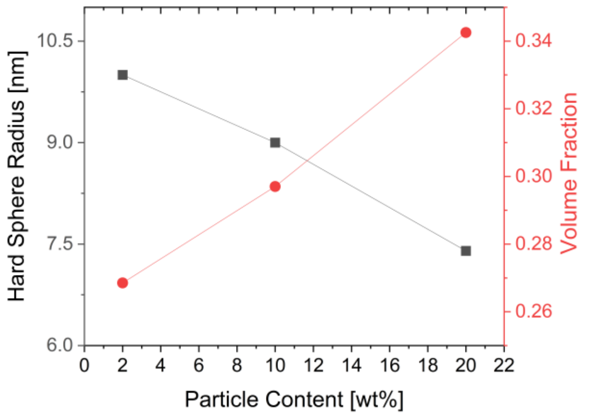

Figure S49: Hard-sphere radius (black squares) and volume fraction (red circles) of the composite samples stored under high humidity.

Table S 4: Results of the SAXS measurements of the samples stored at high humidity and after tensile testing.

	Samples with High Water Content			Samples after Tensile Testing		
Parameter	R_{HS}	m	η [%]	R_{HS}	m	η [%]
NC2	10.0	3.0	26.9	10.1	3.1	26.9
NC10	9.0	3.4	29.7	7.8	2.8	31.5
NC20	7.4	2.9	34.3	8.0	3.0	32.6

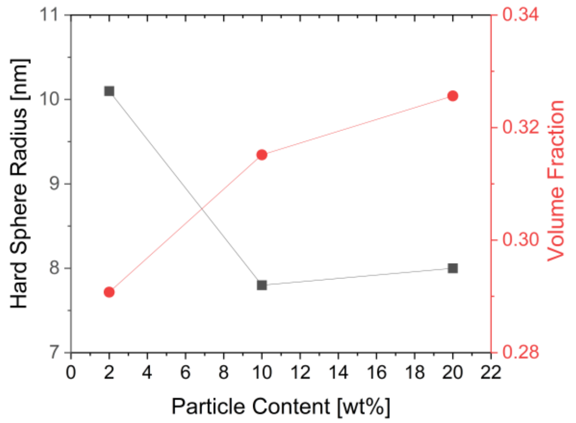

Figure S50: Hard-sphere radius (black squares) and volume fraction (red circles) of the composite samples after tensile testing.

12. Microscopy images of the self-healing tests.

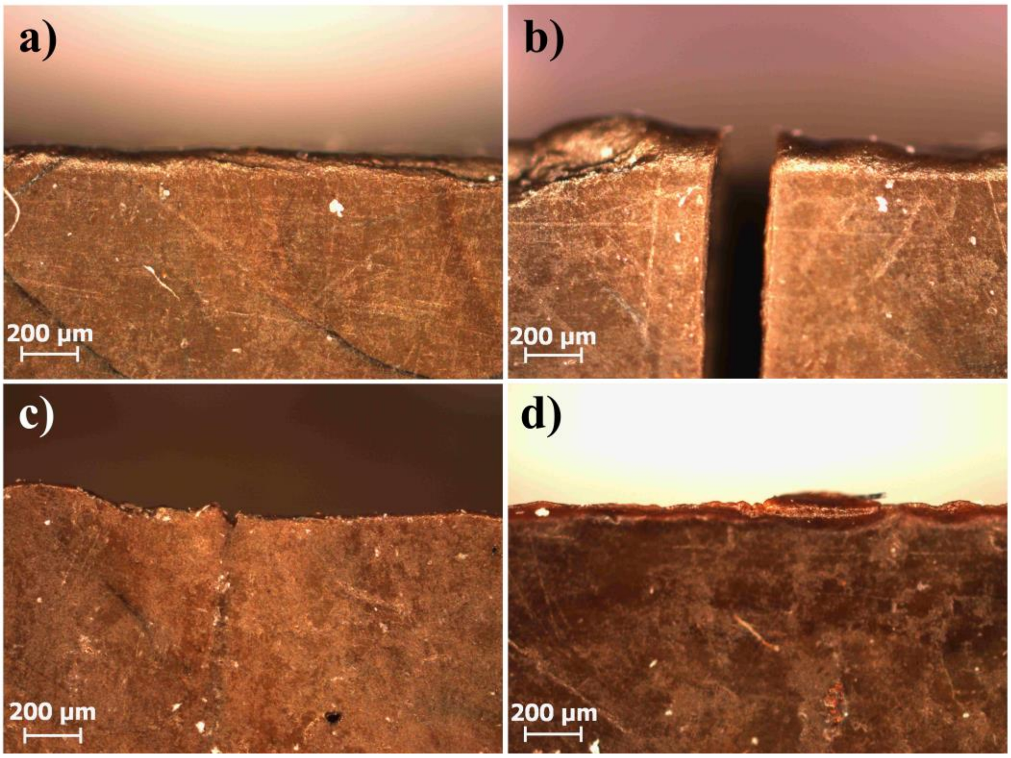

Figure S51: Microscope images of a) NC10, b) cut through half thickness, c) healed at 80 °C for 24 h, and d) another 24 h at 80 °C in a Teflon mold.

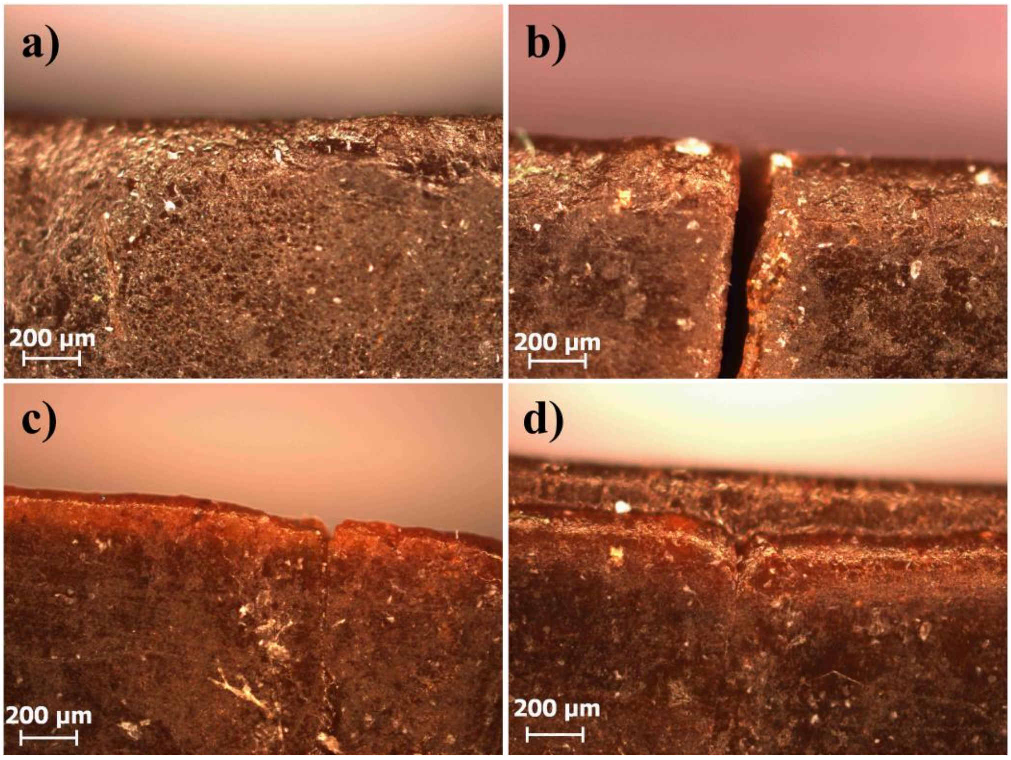

Figure S52: Microscope images of a) NC20, b) cut through half thickness, c) healed at 80 °C for 24 h, and d) another 24 h at 80 °C in a Teflon mold.

References:

[1] Oberhausen, B.; Kickelbick, G. Induction Heating Induced Self-Healing of Nanocomposites *Nanoscale Adv.*, **2021**, *3*, 5589–5604.

[2] Sun, S.; Zeng, H.; Robinson, D.B.; Raoux, S.; Rice, P.M.; Wang, S.X.; Li, G. Monodisperse MFe_2O_4 (M = Fe, Co, Mn) Nanoparticles. *J. Am. Chem. Soc.*, **2004**, *126*, 273–279.

[3] Niederhauser, W.D.; Broderick, E.; Owings, F.F. Method for Preparing Salts of Sulfoalkyl Methacrylates, US3759985A, **1960**.

[4] Minoura, Y.; Ueda, M.; Mizunuma, S.; Oba, M. The Reaction of Polypropylene with Maleic Anhydride. *J. Appl. Polym. Sci.*, **1969**, *13*, 1625–1640.

[5] Stickler, M. Free-Radical Polymerization Kinetics of Methyl Methacrylate at Very High Conversions. *Makromol. Chem.*, **1983**, *184*, 2563–2579.

[6] Saadat, Y.; Kim, K.; Foudazi, R. Initiator-Dependent Kinetics of Lyotropic Liquid Crystal-Templated Thermal Polymerization. *Polym. Chem.*, **2021**, *12*, 2236–2252.

[7] Eisenberg, A. Clustering of Ions in Organic Polymers. A Theoretical Approach. *Macromolecules*, **1970**, *3*, 147–154.
